# The prognostic value of 19S ATPase proteasome subunits in acute myeloid leukemia and other forms of cancer

**DOI:** 10.3389/fmed.2023.1209425

**Published:** 2023-07-12

**Authors:** Boranai Tychhon, Jesse C. Allen, Mayra A. Gonzalez, Idaly M. Olivas, Jonathan P. Solecki, Mehrshad Keivan, Vanessa V. Velazquez, Emily B. McCall, Desiree N. Tapia, Andres J. Rubio, Connor Jordan, David Elliott, Anna M. Eiring

**Affiliations:** ^1^Paul L. Foster School of Medicine, Texas Tech University Health Sciences Center at El Paso, El Paso, TX, United States; ^2^Center of Emphasis in Cancer, Department of Molecular and Translational Medicine, Texas Tech University Health Sciences Center at El Paso, El Paso, TX, United States; ^3^L. Frederick Francis Graduate School of Biomedical Sciences, Texas Tech University Health Sciences Center at El Paso, El Paso, TX, United States

**Keywords:** oncogenes, drug targets, cancer, proteasome inhibition, 19S proteasome

## Abstract

**Introduction:**

The ubiquitin-proteasome system (UPS) is an intracellular organelle responsible for targeted protein degradation, which represents a standard therapeutic target for many different human malignancies. Bortezomib, a reversible inhibitor of chymotrypsin-like proteasome activity, was first approved by the FDA in 2003 to treat multiple myeloma and is now used to treat a number of different cancers, including relapsed mantle cell lymphoma, diffuse large B-cell lymphoma, colorectal cancer, and thyroid carcinoma. Despite the success, bortezomib and other proteasome inhibitors are subject to severe side effects, and ultimately, drug resistance. We recently reported an oncogenic role for non-ATPase members of the 19S proteasome in chronic myeloid leukemia (CML), acute myeloid leukemia (AML), and several different solid tumors. In the present study, we hypothesized that ATPase members of the 19S proteasome would also serve as biomarkers and putative therapeutic targets in AML and multiple other cancers.

**Methods:**

We used data from The Cancer Genome Atlas (TCGA) and the Clinical Proteomic Tumor Analysis Consortium (CPTAC) available at UALCAN and/or GEPIA2 to assess the expression and prognostic value of proteasome 26S subunit, ATPases 1-6 (PSMC1-6) of the 19S proteasome in cancer. UALCAN was also used to associate *PSMC1*-6 mRNA expression with distinct clinicopathological features. Finally, cBioPortal was employed to assess genomic alterations of *PSMC* genes across different cancer types.

**Results:**

The mRNA and protein expression of *PSMC1*-6 of the 19S proteasome were elevated in several cancers compared with normal controls, which often correlated with worse overall survival. In contrast, AML patients demonstrated reduced expression of these proteasome subunits compared with normal mononuclear cells. However, AML patients with high expression of *PSMC2*-5 had worse outcomes.

**Discussion:**

Altogether, our data suggest that components of the 19S proteasome could serve as prognostic biomarkers and novel therapeutic targets in AML and several other human malignancies.

## 1. Introduction

The ubiquitin-proteasome system (UPS) is the key intracellular machinery for protein degradation, regulating the localization and stability of thousands of proteins within a cell (1). The UPS encompasses a series of essential components, including ubiquitin, ubiquitin-activating enzymes (E1s), ubiquitin-conjugating enzymes (E2s), ubiquitin ligases (E3s), deubiquitinating enzymes (DUBs), and the 26S proteasome (2–4). Due to the major role of the UPS in maintaining protein homeostasis, it is a central player in regulating essential cellular functions, including cell differentiation, cell cycle progression, apoptosis, DNA repair, and drug resistance (5–8). The widespread influence of the UPS on cell biology has important implications for human health, as abnormal UPS function is associated with numerous human conditions, including neurodegenerative diseases, autoimmune diseases, and cancer (9, 10). For this reason, a number of small molecule inhibitors targeting the UPS were developed for therapeutic intervention, but unfortunately, drug resistance and toxicity remain significant challenges (11, 12), especially in hematopoietic cells (13–15). Novel therapeutic approaches will be required to effectively target the UPS with less toxicity.

**Figure 1:**
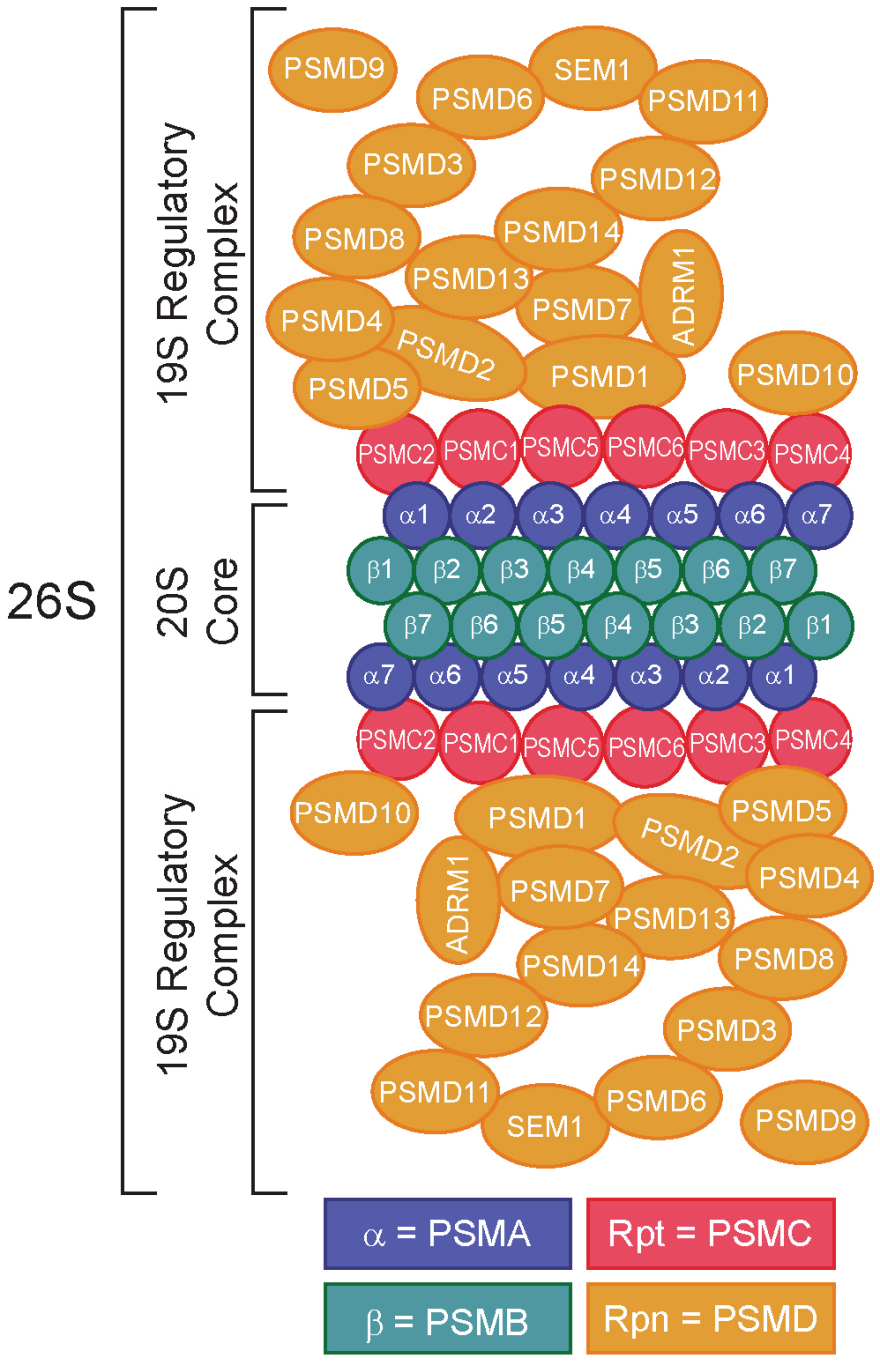
Structure of the 26S proteasome. Subunits of the 20S core particle and 19S regulatory particles are shown. The 20S core complex consists of α1/PSMA1-α7/PSMA7 and β1/PSMB1-β7/PSMB7. The 19S regulatory particles consist of two structures: a base (Rpt1/PSMC1-Rpt6/PSMC6, Rpn1/PSMD2, Rpn2/PSMD1, Rpn10/PSMD10, and Rpn13/ADRM1) and a lid (Rpn3/PSMD3, Rpn4/PSMD10-Rpn9/PSMD13, Rpn11/PSMD14, Rpn12/PSMD8, and Rpn15/SEM1). Rpn4/PSMD9 acts as a chaperone during assembly of the base of the 19S regulatory complex (16). Redrawn from refs (17–21).

The 26S proteasome is comprised of two main subcomplexes, the 20S core complex and the 19S regulatory complex, which work together to recognize ubiquitylated peptides and promote UPS-dependent protein degradation (Figure 1) (22–25). The 20S core complex contains the protein degradative machinery, whereas the 19S regulatory complex functions by recognizing, binding, unfolding, and translocating ubiquitylated peptides into the 20S core complex for degradation (22, 23, 26). Traditional proteasome inhibitors (e.g., bortezomib, carfilzomib, ixazomib) function by binding to and inhibiting the 20S core complex. Bortezomib (Velcade^**®**^), a reversible inhibitor of chymotrypsin-like proteasome activity, acts by binding to and inhibiting the 20S beta 5 subunit of the proteasome (β5, PSMB5) (27), leading to the accumulation of ubiquitylated proteins and cell death (28). However, proteasome inhibitors like bortezomib are prone to resistance mechanisms, due to mutations in the molecular target of bortezomib, PSMB5, highlighting the need for alternative therapeutic strategies (29–31).

One strategy to overcome proteasome inhibitor resistance is to target the 19S regulatory complex instead of the 20S core complex. Importantly, the 19S complex contains numerous components subdivided into ATP-dependent and ATP-independent subunits (Figure 1) (32). The ATP-dependent subunits include the proteasome 26S subunit, ATPases 1–6 (PSMC1-6, Supplementary Table S1), which make up the base of the 19S complex, whereas the ATP-independent subunits include proteasome 26S subunit, non-ATPases 1–16 (PSMD1-16) (25), which make up the 19S lid (33). The lid is important for interactions with ubiquitin, whereas the base is required for unfolding the protein to allow it to enter the 20S core (22). The ATPase subunits play critical roles in substrate engagement, unfolding, translocation, and opening the gate of the core complex (34–37). Several groups have demonstrated the potential for targeting the 19S adhesion-regulating molecule 1 (ADRM1/hRPN13) protein in bortezomib resistance (38–44), leading to the development of the putative ADRM1/hRPN13 inhibitor, RA190 (45–48). Intriguingly, data from our lab and others demonstrated that certain members of the 19S lid might serve as better molecular targets for proteasome inhibition (24, 25, 38, 49–51). Indeed, our research has shown that knockdown of 26S proteasome non-ATPases 1 (PSMD1/hRPN2) and 3 (PSMD3/hRPN3), two members of the 19S regulatory complex (17), impaired survival and induced apoptosis of myeloid leukemia cells but not normal cord blood CD34^+^ hematopoietic stem and progenitor cells (25, 49), implying they may be good molecular targets for proteasome inhibition in human diseases. However, while the PSMD subunits of the 19S proteasome are non-ATPases that do not contain catalytically active target sites, the PSMC subunits are ATPases that could be targeted more efficiently with small molecule inhibitors. Therefore, we hypothesized that ATPase members of the 19S proteasome would serve as biomarkers and putative therapeutic targets in acute myeloid leukemia and several different solid tumors. Using data from The Cancer Genome Atlas (TCGA) and the Clinical Proteomic Tumor Analysis Consortium (CPTAC), expression of the ATPase PSMC subunits of the 19S proteasome was elevated in several cancers compared with normal controls, which often correlated with worse overall survival. Altogether, our data suggest that components of the 19S proteasome could serve as prognostic biomarkers and novel therapeutic targets in multiple human malignancies.

## 2. Materials and methods

### 2.1. Analysis of PSMC differential mRNA and protein expression across TCGA cancers compared with normal tissue

UALCAN (The University of ALabama at Birmingham CANcer data analysis portal)^1^ is a comprehensive, interactive platform for in-depth analyses of TCGA and CPTAC data (52). This portal helped us identify potential candidates based on mRNA and protein expression data across different tumor stages and cancer types compared with normal tissue. The cancers we investigated included acute myeloid leukemia (LAML), adrenocortical carcinoma (ACC), bladder urothelial carcinoma (BLCA), breast invasive carcinoma (BRCA), cervical squamous cell carcinoma and endocervical adenocarcinoma (CESC), cholangiocarcinoma (CHOL), colorectal adenocarcinoma (COAD), diffuse large B-cell lymphoma (DLBCL), esophageal carcinoma (ESCA), glioblastoma multiforme (GBM), head and neck squamous cell carcinoma (HNSC), kidney chromophobe (KICH), kidney renal clear cell carcinoma (KIRC), kidney renal papillary cell carcinoma (KIRP), liver hepatocellular carcinoma (LIHC), low-grade glioma (LGG), lung adenocarcinoma (LUAD), lung squamous cell carcinoma (LUSC), mesothelioma (MESO), ovarian serous cystadenocarcinoma (OV), pancreatic adenocarcinoma (PAAD), pheochromocytoma and paraganglioma (PCPG), prostate adenocarcinoma (PRAD), rectum adenocarcinoma (READ), sarcoma (SARC), skin cutaneous melanoma (SKCM), stomach adenocarcinoma (STAD), testicular germ cell tumors (TGCT), thymoma (THYM), thyroid carcinoma (THCA), uterine carcinosarcoma (UCS), uterine corpus endometrial carcinoma (UCEC), and uveal melanoma (UVM). The comparison of mRNA expression for *PSMC* subunits between tumor and normal tissues was further studied using GEPIA2 (Gene Expression Profiling Interactive Analysis 2)^2^, an integrated web-based platform for analyzing gene expression data from TCGA and the Genotype-Tissue Expression (GTEx) projects (53). Data are presented using box plots.

### 2.2. Correlation of *PSMC* mRNA expression with prognostic significance across different cancer types

Along with the gene expression analyses described above, we also used UALCAN to correlate gene expression levels for *PMSC1-6* with overall survival across TCGA cancers. Data are presented using Kaplan–Meier curves.

### 2.3. Correlation of *PSMC* mRNA expression with distinct clinicopathological features in different cancer types

UALCAN also provided the mRNA expression of *PSMC* subunits across cancer types comparing different cancer stages. We chose to focus on the expression of *PSMC1-6* across tumor stages for patients with BRCA, LIHC, KICH, and LUAD. Additionally, we assessed *PSMC1-6* expression in AML patients with mutated versus unaltered FMS-like tyrosine kinase 3 (FLT3) using data from UALCAN and the BEAT AML trial (ClinicalTrials.gov Identifier: NCT03013998) available at Vizome^3^ (54, 55).

### 2.4. Genomic alterations of PSMC genes across different cancer types

cBioPortal^4^ is another online database for cancer genomics used to explore and visualize genomic data across cancer studies (56). Therefore, we analyzed the frequency and different types of genetic mutations in *PSMC* subunit genes across different human cancers.

### 2.5. Statistical analyzes

Differences in PSMC gene and protein expression levels were calculated in UALCAN using the Student’s *t*-test, whereas GEPIA2 data were analyzed using the one-way ANOVA test. Data are presented using box plots. Correlations of gene expression with overall survival are presented using Kaplan–Meier curves (UALCAN uses a 25% cutoff for analysis of survival data). A value of *p* of <0.05 was considered statistically significant for all analyzes.

## 3. Results

### 3.1. mRNA encoding proteasome subunits were often downregulated in AML versus normal progenitors, but patients with higher expression levels had a worse prognosis

Work from our lab and others had documented increased expression of several 19S proteasome subunits in multiple cancer types compared with normal tissue (24, 49, 51, 57–60). Our previous work in AML studying the non-ATPase subunits of the 19S proteasome revealed that *PSMD2*, *PSMD3*, *PSMD7*, and *PSMD9* mRNA expression levels were reduced in AML versus normal progenitors, but that high levels of expression correlated with worse overall survival in AML patients (25). In fact, the downregulation of proteasome subunits is a universal feature of AML compared with normal mononuclear cells. For the 20S proteasome, *PSMA4*, *PSMA5*, *PSMA7*, and all seven *PSMB* subunits are downregulated in AML versus normal controls (Supplementary Figure S1). However, similar to our observations for *PSMD3* (25), patients with higher expression levels tended to have worse outcomes (Supplementary Figures S2, S3).

**Figure 2:**
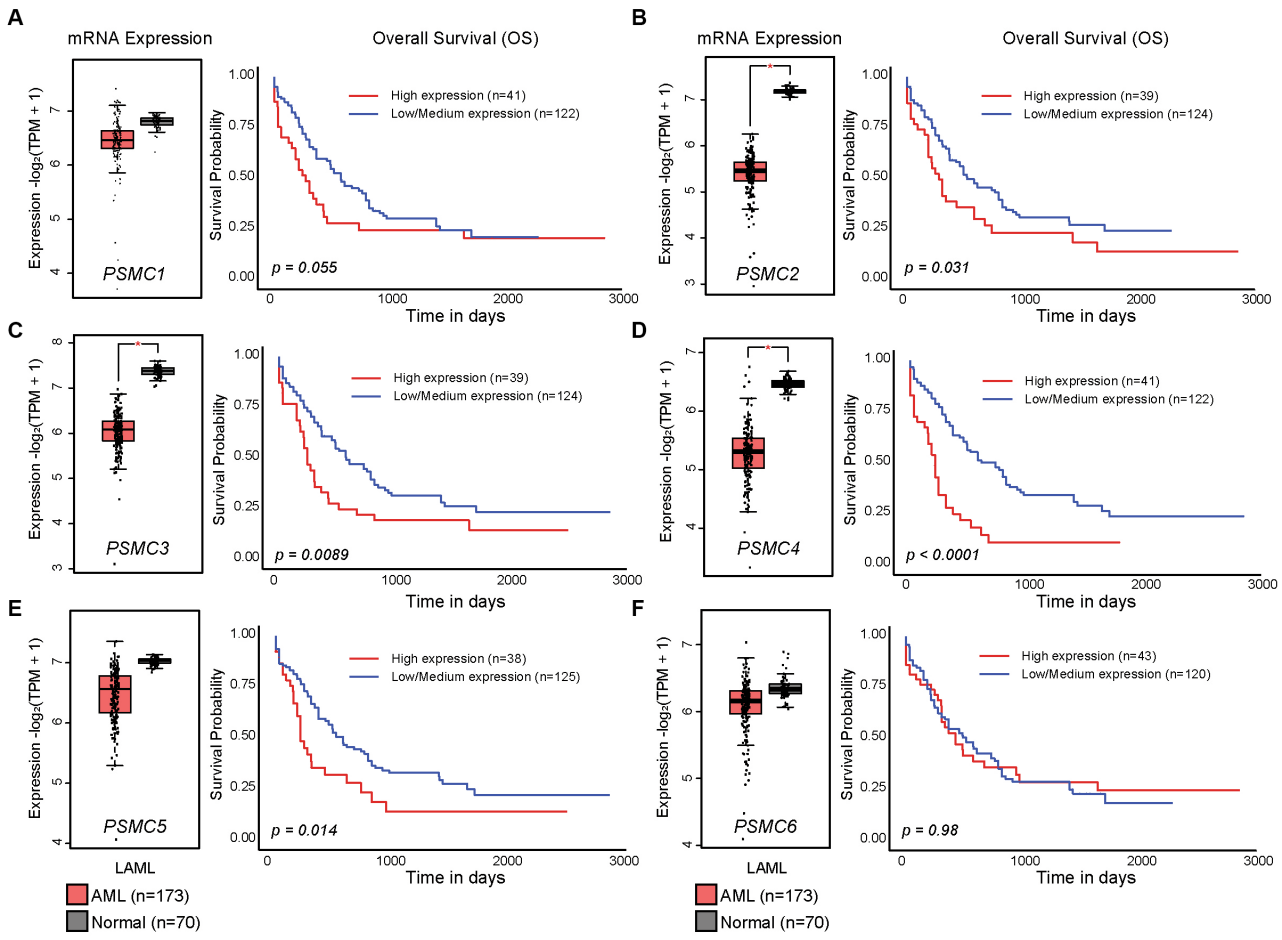
High levels of *PSMC* subunit expression correlated with worse overall survival in acute myeloid leukemia (AML). **(A–F)**. Box plots (*left*) and Kaplan–Meier curves (*right*) show the expression of *PSMC1* **(A)**, *PSMC2* **(B)**, *PSMC3* **(C)**, *PSMC4* **(D)**, *PSMC5* **(E)**, and *PSMC6* **(F)** mRNA in AML versus normal specimens and their correlation with overall survival. *p < 0.001.

In the present study, we hypothesized that ATPase members of the 19S proteasome would show a similar expression pattern, and that is indeed what we observed. *PSMC2*, *PSMC3*, and *PSMC4* mRNA expression were significantly reduced in AML versus normal progenitor cells, whereas no change was observed for *PSMC1*, *PSMC5*, or *PSMC6* (Figures 2A–F, *left*). These data were confirmed using the GEPIA2 database (Supplementary Figure S4). Despite this observation, TCGA data available at UALCAN showed that AML patients with higher than average levels of *PSMC2*, *PSMC3*, *PSMC4*, and *PSMC5* mRNA expression had a significant reduction in overall survival compared with patients expressing low to medium levels of those genes when all AML subtypes were considered (Figures 2A–F, *right*).

**Figure 3:**
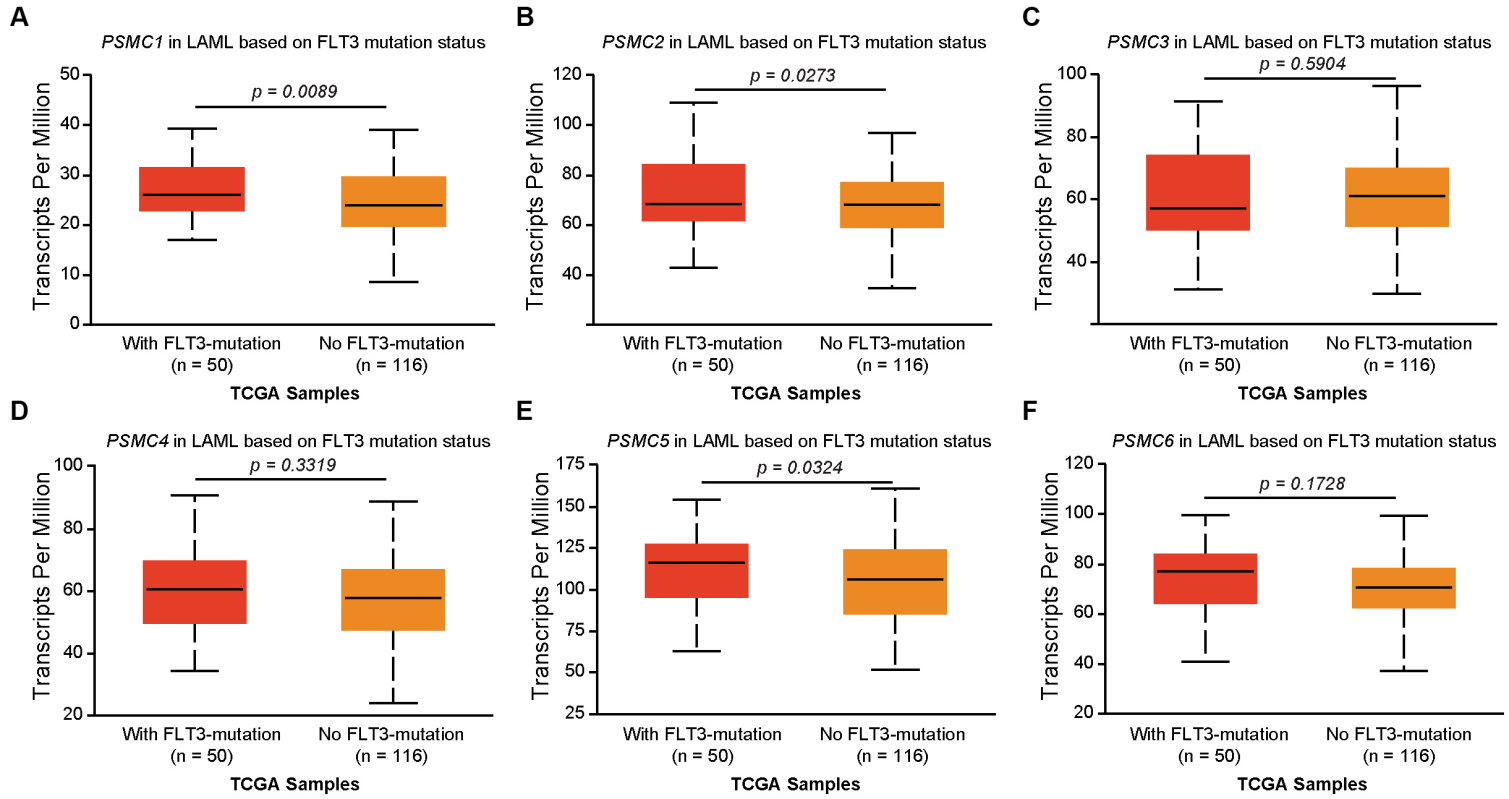
Correlation of *PSMC* subunit expression with FLT3 mutation status in AML. **(A–F)** We used TCGA data available at UALCAN to associate the expression of *PSMC* subunits with FLT3 mutation status in AML. The box plots demonstrate *PSMC1* **(A)**, *PSMC2* **(B)**, *PSMC3* **(C)**, *PSMC4* **(D)**, *PSMC5* **(E)**, and *PSMC6* **(F)** mRNA expression comparing AML patients with mutated versus wild-type FLT3.

**Figure 4:**
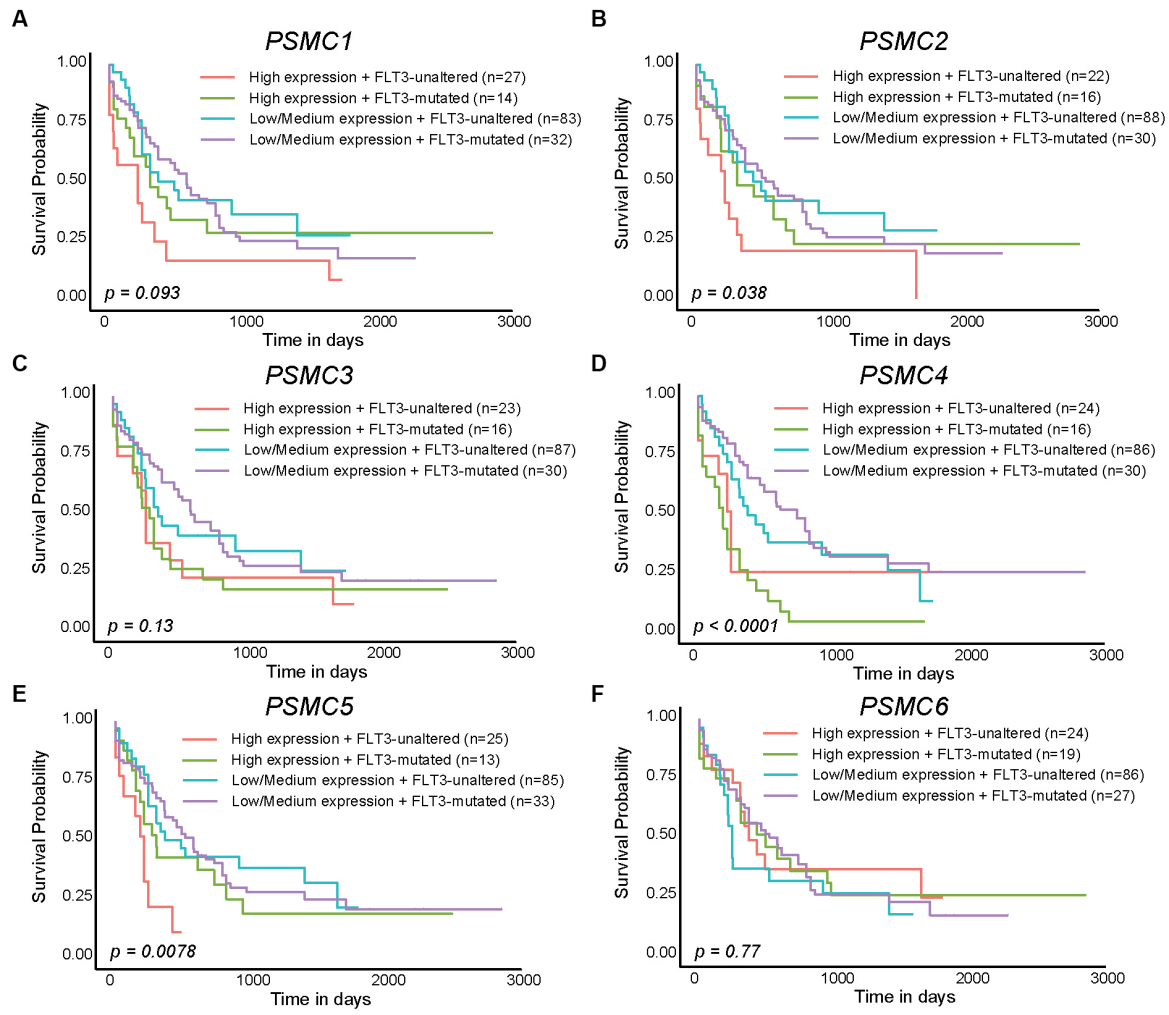
High levels of *PSMC2*, *PSMC4*, and *PSMC5* mRNA expression correlated with worse outcomes in AML patients with mutated and/or wild-type FLT3. **(A–F)**. Kaplan–Meier curves show the effects of *PSMC1* **(A)**, *PSMC2* **(B)**, *PSMC3* **(C)**, *PSMC4* **(D)**, *PSMC5* **(E)**, and *PSMC6* **(F)** mRNA expression on overall survival comparing AML patients with mutated versus wild-type (unaltered) FLT3.

We previously demonstrated that high levels of *PSMD3* mRNA expression led to a sharp reduction in overall survival for AML patients harboring *FLT3* mutations (25). We next asked whether the expression of ATPase subunits of the 19S proteasome was altered in AML patients with mutated versus wild-type FLT3 and whether they correlated with overall survival. TCGA data available at UALCAN demonstrated that the genes encoding *PSMC1* (*p* = 0.009), *PSMC2* (*p* = 0.027), and *PSMC5* (*p* = 0.032) were significantly upregulated in AML patients with mutated versus wild-type FLT3 (Figures 3A–F). *PSMC5* upregulation in FLT3+ AML was confirmed using data from the BEAT AML trial available at Vizome.org (Supplementary Figure S5) (54, 55). Consistent with a potential role for these proteins in drug resistance of AML, patients with high levels of *PSMC2*, *PSMC4*, and *PSMC5* mRNA expression had significantly worse overall survival in FLT3-mutated and FLT3-unaltered AML patients (Figures 4A–F). More specifically, high levels of *PSMC4* expression affected both FLT3-mutated (green curve) and FLT3-unaltered patients (pink curve, Figure 4D), whereas patients with high levels of *PSMC2* or *PSMC5* primarily affected AML patients with unaltered FLT3 (pink curves, Figures 4B,E). Altogether, these data implicate PSMC2, PSMC4, and PSMC5, all ATPase subunits of the 19S proteasome, as putative oncogenes in AML, depending on FLT3 status.

### 3.2. Expression of PSMC subunits of the 19S proteasome were elevated at the mRNA and protein levels in multiple solid tumors compared with normal tissue

**Figure 5:**
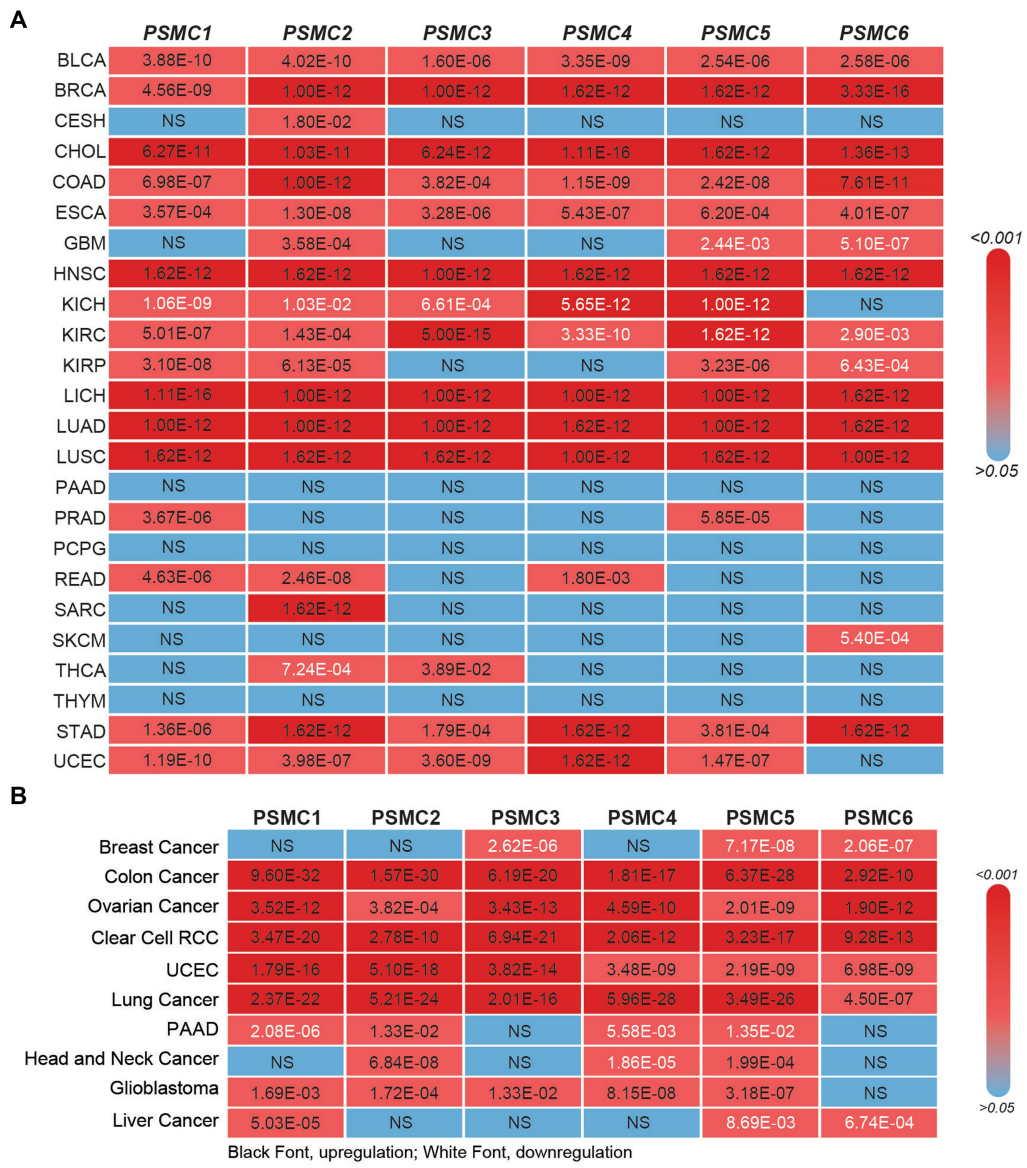
19S PSMC subunit expression at the mRNA and protein level in multiple types of solid tumors. **(A,B)** The heat maps show the significant *p* values comparing PSMC mRNA **(A)** and protein **(B)** levels in all TCGA cancers versus normal specimens, excluding LAML. *p* values indicating significant PSMC upregulation are indicated in black; *p* values indicating significant PSMC downregulation are indicated in white. BLCA, bladder urothelial carcinoma; BRCA, breast invasive carcinoma; CESC, cervical squamous cell carcinoma and endocervical adenocarcinoma; CHOL, cholangiocarcinoma; COAD, colorectal adenocarcinoma; ESCA, esophageal carcinoma; GBM, glioblastoma multiforme; HNSC, head and neck squamous cell carcinoma; KICH, kidney chromophobe; KIRC, kidney renal clear cell carcinoma; KIRP, kidney renal papillary cell carcinoma; LIHC, liver hepatocellular carcinoma; LUAD, lung adenocarcinoma; LUSC, lung squamous cell carcinoma; PAAD, pancreatic adenocarcinoma; PRAD, prostate adenocarcinoma; PCPG, pheochromocytoma and paraganglioma; RCC, renal cell carcinoma; READ, rectum adenocarcinoma; SARC, sarcoma; SKCM, skin cutaneous melanoma; THCA, thyroid carcinoma; THYM, thymoma; STAD, stomach adenocarcinoma; UCEC, uterine corpus endometrial carcinoma.

Prior studies have demonstrated that the expression of several different *PSMC* proteasome subunits is increased in lung cancer, breast cancer, and multiple myeloma (58–61). Therefore, we hypothesized that the relevance of PSMC 19S proteasome subunits might stretch beyond AML to other types of cancers. At the mRNA level, TCGA data available at UALCAN demonstrated that the expression of *PSMC1* (15/24 tumors, 62.5%), *PSMC2* (17/24 tumors, 70.8%), *PSMC3* (13/24 tumors, 54.2%), *PSMC4* (12/24 tumors, 50%), *PSMC5* (13/24 tumors, 54.2%), and *PSMC6* (10/24 tumors, 41.7%) mRNA were significantly elevated in several different tumor types compared with normal tissue (Figure 5A; Supplementary Figures S6, S7). For instance, BLCA, BRCA, CHOL, COAD, ESCA, HNSC, LICH, LUAD, LUSC, and STAD cancers demonstrated increased expression of all six *PSMC* subunits (Figure 5A; Supplementary Figures S6, S7). On the other hand, patients with glioblastoma (GBM) showed increased expression of *PSMC2*, but decreased expression of *PSMC5* and *PSMC6*. Patients with kidney chromophobe (KICH) were the marked exception, demonstrating reduced expression of *PSMC1*, *PSMC2*, *PSMC3*, *PSMC4*, and *PSMC5* in tumor versus normal tissues (Figure 5A; Supplementary Figures S6, S7).

Similar to the mRNA data, CPTAC data available at UALCAN revealed that the expression of PSMC1 (6/10 tumors, 60%), PSMC2 (8/10 tumors, 80%), PSMC3 (6/10 tumors, 60%), PSMC4 (6/10 tumors, 60%), PSMC5 (6/10 tumors, 60%), and PSMC6 (5/10 tumors, 50%) proteins were significantly elevated in several different tumor types compared with normal tissue (Figure 5B; Supplementary Figures S8, S9). Importantly, patients with colon cancer, ovarian cancer, clear cell renal cell carcinoma (RCC), UCEC, and lung cancer demonstrated increased expression of all six PSMC protein subunits. On the other hand, patients with breast cancer, who presented increased expression of all six *PSMC* mRNAs, showed a significant reduction of PSMC3, PSMC5, and PSMC6 protein levels. Patients with PAAD or liver cancer also demonstrated reduced expression of PSMC protein subunits (Figure 5B; Supplementary Figures S8, S9). Altogether, these data further implicate the ATPase subunits of the 19S proteasome as oncogenes in AML and other types of cancers.

### 3.3. High expression of *PSMC* subunits correlated with worse outcomes in a number of different human malignancies

**Figure 6:**
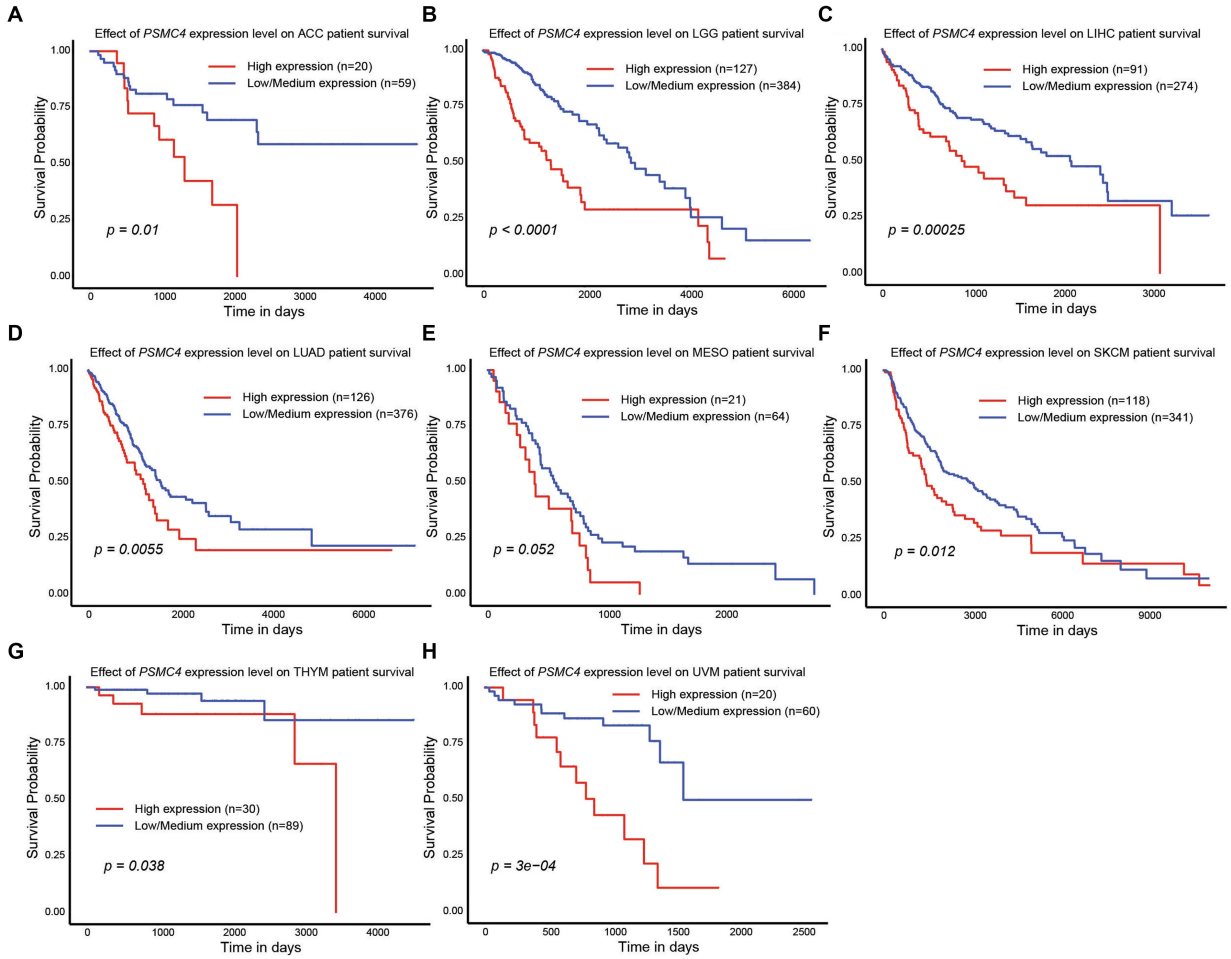
*PSMC4* expression correlated with worse overall survival in multiple human malignancies. **(A–H)** TCGA data available at UALCAN demonstrate that higher levels of *PSMC4* mRNA expression correlated with worse outcomes for adrenocortical carcinoma ACC **(A)**, brain lower grade glioma LGG, **(B)** liver hepatocellular carcinoma LIHC, **(C)** lung adenocarcinoma LUAD, **(D)** mesothelioma MESO, **(E)** skin cutaneous melanoma SKCM, **(F)** thymoma THYM, **(G)** and uveal melanoma UVM **(H)**.

**Figure 7:**
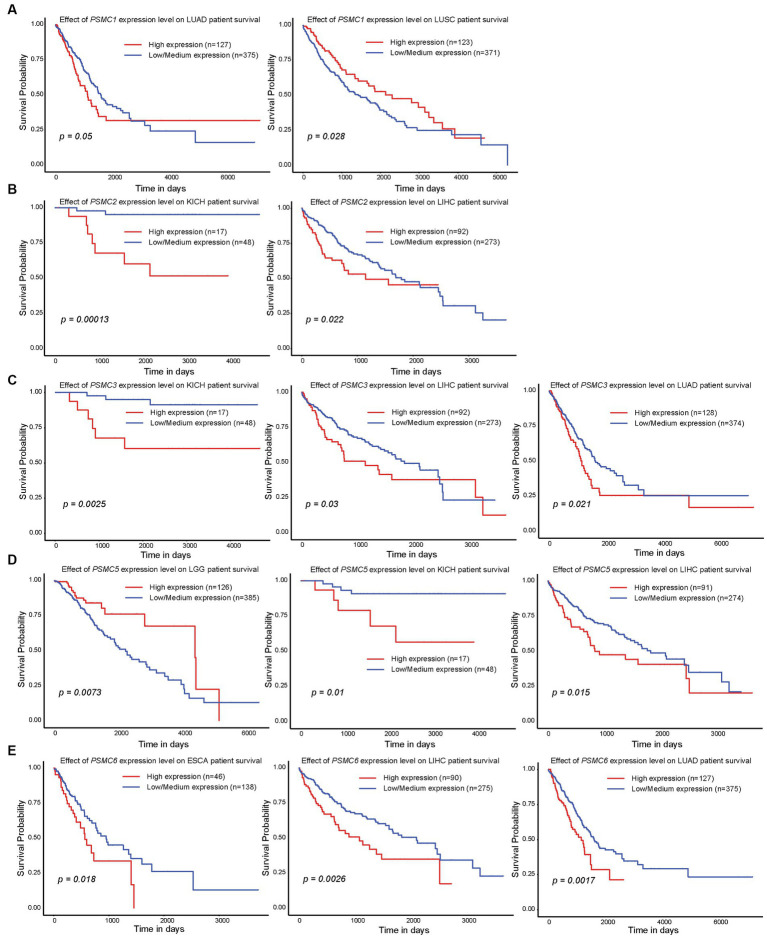
Expression of *PSMC1, 2, 3, 5, and 6* also correlated with changes in overall survival in different solid tumors. **(A–E)** Kaplan–Meier curves show the effects of *PSMC1* **(A)**, *PSMC2* **(B)**, *PSMC3* **(C)**, *PSMC5* **(D)**, and *PSMC6* **(E)** mRNA expression on overall survival in the specified solid tumors. ESCA, esophageal carcinoma; KICH, kidney chromophobe; LGG, brain lower grade glioma; LIHC, liver hepatocellular carcinoma; LUAD, lung adenocarcinoma; LUSC, lung squamous cell carcinoma.

Since high expression of ATPase members of the 19S proteasome correlated with worse outcomes in AML (Figures 2, 4), we hypothesized that similar results would be observed in other types of cancers. All six ATPase subunits of the 19S proteasome correlated with worse overall survival in multiple human malignancies, but the expression of *PSMC4* appeared to have the greatest effect (Figure 6). High levels of *PSMC4* mRNA expression yielded worse outcomes in eight different malignancies, including patients with ACC (*p* = 0.01), LGG (*p* < 0.0001), LICH (*p* = 0.00025), LUAD (*p* = 0.0055), MESO (*p* = 0.052), SKCM (*p* = 0.012), THYM (*p* = 0.038), and UVM (*p* = 0.0003; Figures 6A–H). While AML patients with high *PSMC1* expression had a worse overall survival (Figures 2, 4), *PSMC1* mRNA expression had little effect on outcomes in solid tumors, except for patients with LUAD and LUSC. Interestingly, high levels of *PSMC1* mRNA expression correlated with worse outcomes in LUAD (*p = 0.05*), but better outcomes in LUSC (*p* = 0.028; Figure 7A). Similarly, patients with high levels of *PSMC2* expression demonstrated reduced overall survival for patients with KICH (*p* = 0.00013) and LIHC (*p* = 0.022; Figure 7B). High levels of PSMC3 expression correlated with worse outcomes for patients with KICH (*p* = 0.0025), LIHC (*p* = 0.03), and LUAD (*p* = 0.021; Figure 7C). High levels of *PSMC5* correlated with better outcomes in patients with LGG (*p* = 0.0073), but worse overall survival in patients with KICH (*p* = 0.01) and LIHC (*p* = 0.015; Figure 7D). Finally, high levels of *PSMC6* significantly reduced overall survival in patients with ESCA (*p* = 0.018), LIHC (*p* = 0.0026), and LUAD (*p* = 0.0017; Figure 7E). Altogether, ATPase members of the 19S proteasome were frequently upregulated in multiple types of cancers, which correlated with worse outcomes.

### 3.4. Correlation of *PSMC* subunit expression with distinct clinicopathological features in patients with BRCA, KICH, LIHC, and LUAD

**Figure 8:**
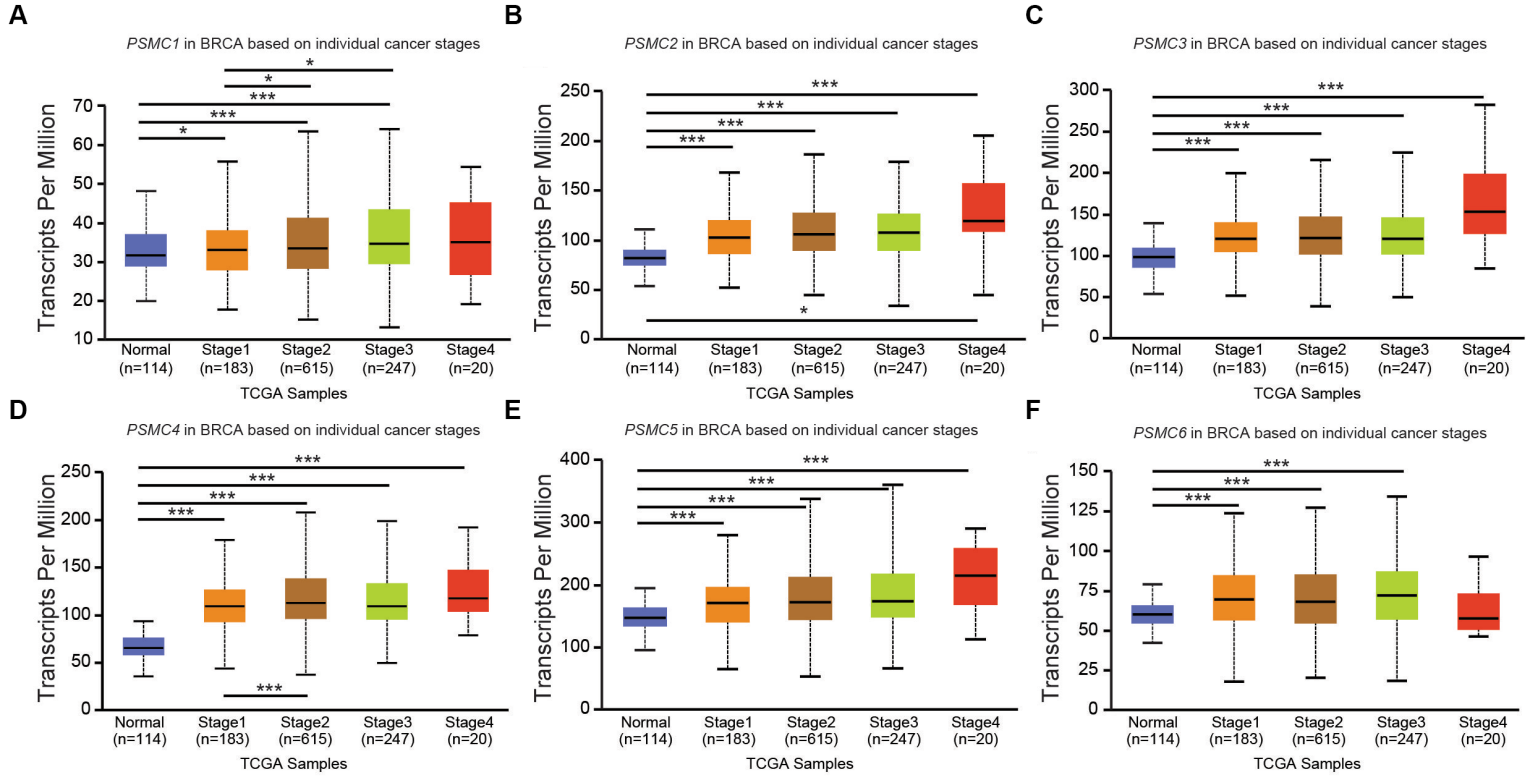
*PSMC* subunit expression correlated with tumor staging in breast invasive carcinoma (BRCA). **(A–F)** The box plots represent *PSMC1* **(A)**, *PSMC2* **(B)**, *PSMC3* **(C)**, *PSMC4* **(D)**, *PSMC5* **(E)**, and *PSMC6* **(F)** mRNA expression in stages 1–4 of BRCA compared with normal samples from The Cancer Genome Atlas (TCGA).

**Figure 9:**
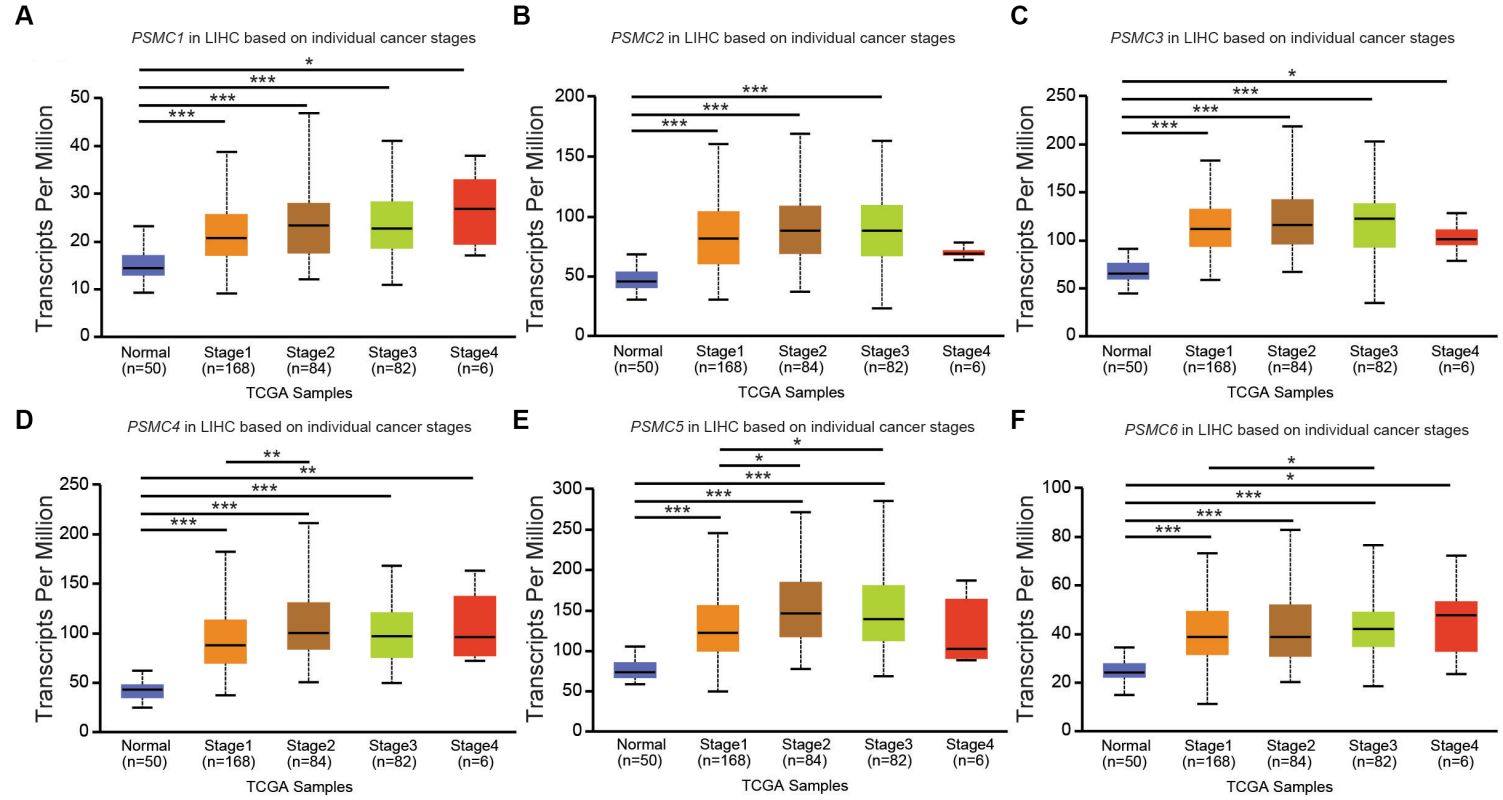
*PSMC* subunit expression correlated with tumor staging in liver hepatocellular carcinoma (LIHC). **(A–F)** The box plots represent *PSMC1* **(A)**, *PSMC2* **(B)**, *PSMC3* **(C)**, *PSMC4* **(D)**, *PSMC5* **(E)**, and *PSMC6* **(F)** mRNA expression in stages 1–4 of LIHC compared with normal samples from The Cancer Genome Atlas (TCGA). *p < 0.05; **p < 0.01; ***p < 0.001.

According to data from UALCAN, the mRNA expression of *PSMC* subunits differs significantly among different tumor stages in certain types of cancers. In BRCA, for example, expression of *PSMC2*, *PSMC3*, and *PSMC5* were elevated in stages 1–4 compared with normal tissues, with the highest levels observed at stage 4 (Figures 8A–F). Interestingly, *PSMC1* expression increased in BRCA patients who progressed from stage 1 to stages 2–3 of the disease (Figure 8A). In KICH, on the other hand, the expression levels of all *PSMC* subunits were significantly reduced in stages 1, 2, and 3 compared with normal tissues (*p* < 0.05; Supplementary Figures S10A–F). However, with the exception of *PSMC1*, a notable feature in KICH is a distinct upregulation of *PSMC2-6* subunit expression during stage 4 of the disease (Supplementary Figures S10B–F). In LIHC, expression of all six *PSMC* subunits was elevated in stages 1–4 compared with normal tissues. *PSMC4*, *PSMC5*, and *PSMC6* expression increased in patients progressing from stage 1 to stages 2–3 of the disease (Figures 9A–F). In contrast, while all six *PSMC* subunits were elevated in stages 1–4 compared with normal tissue in patients with LUAD, expression was not changed when comparing different stages of the disease (Supplementary Figure S11). These differences in the pattern of ATPase expression suggest a potential method for staging BRCA, KICH, LIHC, and possibly other tumors to provide prognostic information.

### 3.5. Genomic alterations of *PSMC* subunits in cancer

**Figure 10:**
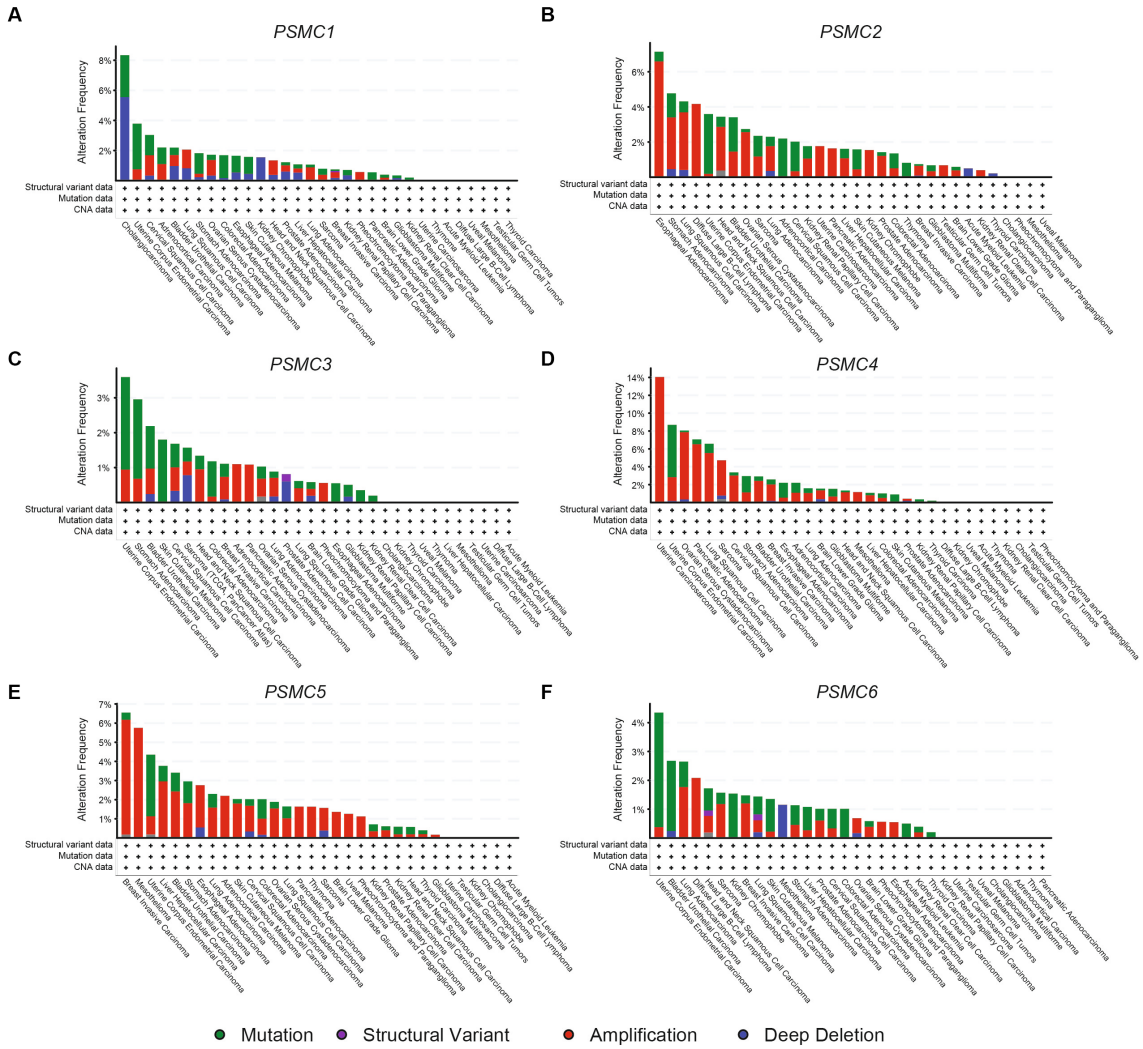
Genomic alterations in the genes encoding PSMC1-6 in multiple human cancers. Using data from cBioPortal, the bar graphs show genomic alterations associated with *PSMC1* **(A)**, *PSMC2* **(B)**, *PSMC3* **(C)**, *PSMC4* **(D)**, *PSMC5* **(E)**, and *PSMC6* **(F)** in all of the cancers available in TCGA. ACC, adrenocortical carcinoma; BLCA, bladder urothelial carcinoma; BRCA, breast invasive carcinoma; CESC, cervical squamous cell carcinoma and endocervical adenocarcinoma; CHOL, cholangiocarcinoma; COAD, colorectal adenocarcinoma; DLBCL, diffuse large B-cell lymphoma; ESCA, esophageal carcinoma; GBM, glioblastoma multiforme; HNSC, head and neck squamous cell carcinoma; KICH, kidney chromophobe; KIRC, kidney renal clear cell carcinoma; KIRP, kidney renal papillary cell carcinoma; LAML, acute myeloid leukemia; LGG, brain lower grade glioma; LIHC, liver hepatocellular carcinoma; LUAD, lung adenocarcinoma; LUSC, lung squamous cell carcinoma; MESO, mesothelioma; OV, ovarian serous cystadenocarcinoma; PAAD, pancreatic adenocarcinoma; PRAD, prostate adenocarcinoma; PCPG, pheochromocytoma and paraganglioma; READ, rectum adenocarcinoma; SARC, sarcoma; SKCM, skin cutaneous melanoma; STAD, stomach adenocarcinoma; TGCT, testicular germ cell tumor; THCA, thyroid carcinoma; THYM, thymoma; UCEC, uterine corpus endometrial carcinoma; UCS, uterine carcinosarcoma; UVM, uveal melanoma.

To better understand the causes of these *PSMC* gene expressions changes in cancer, we used cBioPortal to identify the genomic mutations identified through TCGA. AML showed deep deletions for *PSMC2* and mutations in *PSMC6*, with no other abnormalities in the other subunits (Figures 10A–F). *PSMC1* DNA is prone to mutations, amplifications, and deep deletions, with mutations documented as the most common change (Figure 10A). On the other hand, *PSMC2*, *PSMC4*, and *PSMC5* are primarily prone to amplifications, whereas *PSMC3* and *PSMC6* are more likely to have a combination of mutations and amplifications (Figures 10B–F). Notably, KICH has deep deletions for *PSMC1*, amplifications for *PSMC2*, and mutations for *PSMC6*, with no other abnormalities observed in the other subunits (Figures 10A–F). Altogether, mutations, deep deletions, and amplifications could explain the differences in *PSMC* gene expression in certain types of cancers.

## 4. Discussion

The ubiquitin-proteasome system (UPS) is a multi-protein complex that plays a critical role in regulating protein homeostasis, apoptosis, and a number of other cellular processes. Cancers can have abnormally increased proteasome activity due to the high metabolic demands for cancer growth that require rapid protein turnover. Therefore, the UPS has become a spotlight in research as a therapeutic target for various cancers. Standard proteasome inhibitors bind to and inhibit the 20S core complex of the UPS (27). However, proteasome inhibitors like bortezomib are prone to resistance mechanisms, highlighting the need for alternative therapeutic strategies (29–31). One strategy to overcome proteasome inhibitor resistance is to target the 19S regulatory complex instead of the 20S core complex. Indeed, knockdown of the 19S PSMD3 subunit significantly impaired the stability of human epidermal growth factor 2 (HER2) in breast cancer, which induced apoptosis and inhibited the growth, proliferation, and colony formation of tumor cells. Furthermore, a high level of *PSMD3* mRNA expression was associated with shorter overall survival in breast cancer, especially in HER2+ patients (51). Similarly, our data demonstrated that PSMD3 expression is highly upregulated in chronic myeloid leukemia (CML) patients who have progressed from the chronic to the rapidly fatal blast phase of the disease (49). In marked contrast, however, patients with AML demonstrated reduced expression of *PSMD3* mRNA compared with normal specimens (25). Despite this observation, AML patients with higher than average levels of *PSMD3* expression had worse outcomes, especially for patients with FLT3 mutations (25). Importantly, PSMD3 knockdown impaired survival and induced apoptosis of CML and AML cells, but not normal cord blood progenitors (25, 49), indicating it may be a good potential target for cancer therapy.

The PSMD subunits of the 19S proteasome are non-ATPases that harbor no catalytically active target sites, which may make them poor cancer drug targets. On the other hand, the PSMC subunits of the 19S proteasome are ATPases that harbor catalytically active target sites. Indeed, proton pump inhibitors, also known as H+/K+ ATPase modulators, have attracted much attention for their clinical implication in gastric acid-related diseases (62, 63), whereas vacuolar ATPase inhibitors are being studied as a potential treatment for therapy-resistant cancers (64). Therefore, we hypothesized that ATPase members of the 19S proteasome would serve as biomarkers of disease progression and possible therapeutic targets in AML and multiple solid tumors, and this was indeed what we observed. Similar to our observations for *PSMD3* (25), mRNA encoding PSMC2, PSMC3, and PSMC4 were surprisingly downregulated in AML versus normal mononuclear cells (Figure 2). We speculate that low proteasome subunit expression may explain the suboptimal response of AML patients to proteasome inhibition in certain clinical trials (65). Despite the reduced subunit expression, AML patients with high levels of *PSMC2*, *PSMC3*, *PSMC4*, and *PSMC5* expression had a worse overall survival compared with patients demonstrating low-to-medium levels of expression (Figure 2). Interestingly, *PSMC1*, *PSMC2*, and *PSMC5* mRNA expression were significantly higher in AML patients with mutated versus wild-type FLT3 (Figure 3), which correlated with worse outcomes in the case of *PSMC2* and *PSMC5* (Figure 4). In the case of solid tumors, all six PSMC subunits were upregulated at the mRNA and/or protein level in multiple different cancers, which in some cases correlated with worse outcomes (Figures 5–7). DNA mutations, deep deletions, or amplifications may explain the differences in *PSMC* gene expression observed in certain types of cancers.

As mentioned previously, high expression of all six ATPase subunits of the 19S proteasome correlated with worse overall survival in multiple human malignancies. However, the expression of *PSMC4* appeared to have the greatest effect, correlating with reduced overall survival in 8/24 TCGA tumors (Figure 6). The first evidence for an oncogenic role of PSMC4 was in prostate cancer. In that study, expression of the genes encoding PSMC4, PSMB5, and the E3 ubiquitin ligase NEDD4L, were significantly upregulated in prostate cancer cells compared with the corresponding adjacent normal prostate tissue (66). PSMC4 was also shown to facilitate interactions between the proteasome and the endoplasmic reticulum, allowing for cellular ubiquitination of specific mitochondrial proteins (67). Interestingly, this association was shown to favor the resistance of breast cancer cells to anthracycline treatments (68). Additionally, the proteasome is dynamically phosphorylated during the cell cycle at threonine 25 of the 19S PSMC4 subunit, which contributes to cell proliferation and tumorigenesis (37). Through a kinome-wide screen, they identified dual-specificity tyrosine-regulated kinase 2 (DYRK2) as the primary kinase that phosphorylates PSMC4, leading to enhanced peptide translocation and degradation (37). Genome editing or small-molecule mediated inhibition of DYRK2 significantly bypassed bortezomib resistance in both multiple myeloma and breast cancer cells (69). PSMC4 is also considered a biomarker in endometrial cancer (70–72), breast cancer (60), hepatocellular carcinoma (73), oral squamous cell carcinoma (74), and laryngeal carcinoma (75). Combined with our data in the present study, PSMC4 may be a novel biomarker and therapeutic target for drug-resistant cancer patients demonstrating high levels of PSMC4 expression.

Consistent with our findings, higher proteasome activity has been detected in colon cancer tissue compared with the surrounding normal tissue (76). Furthermore, PSMC2-6 were shown to be more highly expressed at the mRNA and protein level in breast cancer compared with normal breast tissue, which again correlated with worse outcomes (60). Proteasome inhibitors like bortezomib, carfilzomib, or ixazomib have been very effective in the treatment of multiple myeloma, but resistance to therapy is still a major obstacle (13–15, 27, 30). These drugs target the PSMB5 subunit of the 20S proteasome, and mutations in PSMB5 can confer drug resistance in *in vitro* models (31). However, an analysis of 1,500 multiple myeloma patients revealed that these mutations were rare in the clinic (31). A recent meta-analysis study investigated the potential role of PSMC subunits in multiple myeloma and the correlation of mRNA expression with proteasome inhibitor resistance. This analysis included approximately 2000 newly diagnosed and advanced multiple myeloma patients, demonstrating 36 single nucleotide variants in the ATP/ADP binding pockets, which affected the conformation of individual subunits and the structure of the proteasome as a whole. For instance, an acquired mutation of PSMC2 at Y429S after bortezomib therapy correlated with relapse, thereby confirming an association of PSMC2 with proteasome inhibitor resistance (61). Another study identified 19S proteasome subunits as key determinants of resistance to proteasome inhibitors. This study performed a bortezomib screening on the KBM7 human myeloid leukemia cell line, revealing that PSMC2-6 served as key players in proteasome inhibitor resistance, and that downregulation of PSMC5 increased proteasome inhibitor resistance (77). Combined with our data, the PSMC subunits of the 19S proteasome could be novel prognostic biomarkers and putative therapeutic targets in AML and multiple types of solid tumors. These novel cancer drug targets are worthy of future investigation in the fields of cancer therapy and drug resistance.

## Data availability statement

The original contributions presented in the study are included in the article/Supplementary material, further inquiries can be directed to the corresponding author.

## Author contributions

BT: data curation, investigation, methodology, writing-original draft, writing-review and editing, visualization, and formal analysis. JA: data curation, investigation, methodology, writing-original draft, writing-review and editing, visualization, and formal analysis. MG and IO: data curation, investigation, visualization, supervision, writing-original draft, and writing-review and editing. JS, MK, VV, EM, DT, AR, CJ, and DE: writing-review and editing. AE: conceptualization, methodology, formal analysis, data curation, supervision, validation, writing-original draft, and writing-review and editing. All authors contributed to the article and approved the submitted version.

## Funding

This work was funded by the Elsa U. Pardee Foundation (AE), the Scholarly Activity and Research Program (SARP) at Paul L. Foster School of Medicine, and the Department of Molecular and Translational Medicine at Texas Tech University Health Sciences Center at El Paso.

## Conflict of interest

The authors declare that the research was conducted in the absence of any commercial or financial relationships that could be construed as a potential conflict of interest.

## Publisher’s note

All claims expressed in this article are solely those of the authors and do not necessarily represent those of their affiliated organizations, or those of the publisher, the editors and the reviewers. Any product that may be evaluated in this article, or claim that may be made by its manufacturer, is not guaranteed or endorsed by the publisher.

## Footnotes

^1^http://ualcan.path.uab.edu/, accessed on January 12, 2023.

^2^http://gepia2.cancer-pku.cn, accessed on January 12, 2023.

^3^http://vizome.org, accessed on March 22, 2023.

^4^https://www.cbioportal.org, accessed on January 13, 2023.
