# Supplementary material for: The prognostic value of 19S ATPase proteasome subunits in acute myeloid leukemia and other forms of cancer
Source: Front Med (Lausanne). 2023 Jul 12;10:1209425. doi: 10.3389/fmed.2023.1209425 (PMC10371016; doi:10.3389/fmed.2023.1209425)
Supplement: Supplementary file 1 [file Data_Sheet_1.PDF]

frontiers

Supplementary Material

The Prognostic Value of 19S ATPase Proteasome Subunits in Acute Myeloid Leukemia and Other Forms of Cancer

Boranai Tychhon[1#], Jesse C. Allen[1#], Mayra A. Gonzalez[2], Idaly M. Olivas[2], Jonathan P. Solecki[3], Mehrshad Keivan[3], Vanessa V. Velazquez[1], Emily B. McCall[1], Desiree N. Tapia[1], Andres J. Rubio[1], Connor Jordan[3], David Elliott[1], Anna M. Eiring[1,2,3]*

[1]Paul L. Foster School of Medicine, Texas Tech University Health Sciences Center at El Paso, El Paso, TX 79905, USA; [2]Center of Emphasis in Cancer, Department of Molecular and Translational Medicine, Texas Tech University Health Sciences Center at El Paso, El Paso, TX 79905, USA; [3]L. Frederick Francis Graduate School of Biomedical Sciences, Texas Tech University Health Sciences Center at El Paso, El Paso, TX 79905, USA.

[#] These authors contributed equally to this work.

*** Correspondence:** Anna M. Eiring: anna.eiring@ttuhsc.edu

1 Supplementary Tables

Supplementary Table S1. The 19S and 20S subunits of the 26S proteasome

Gene	Alias	Full Name	Location
19S ATPases			
PSMC1	*Rpt2*	Proteasome 26S Subunit, ATPase 1	*chr14:90,256,527-90,275,429*
PSMC2	*Rpt1*	Proteasome 26S Subunit, ATPase 2	*chr7:103,328,570-103,370,346*
PSMC3	*Rpt5*	Proteasome 26S Subunit, ATPase 3	*chr11:47,418,769-47,426,473*
PSMC4	*Rpt3*	Proteasome 26S Subunit, ATPase 4	*chr19:39,971,165-39,981,764*
PSMC5	*Rpt6*	Proteasome 26S Subunit, ATPase 5	*chr17:63,827,152-63,832,026*
PSMC6	*Rpt4*	Proteasome 26S Subunit, ATPase 6	*chr14:52,707,178-52,728,590*
19S non-ATPases			
PSMD1	*Rpn2*	Proteasome 26S Subunit, Non-ATPase 1	*chr2:231,056,845-231,173,116*
PSMD2	*Rpn1*	Proteasome 26S Subunit Ubiquitin Receptor, Non-ATPase 2	*chr3:184,299,198-184,309,050*
PSMD3	*Rpn3*	Proteasome 26S Subunit, Non-ATPase 3	*chr17:39,980,807-39,997,959*
PSMD4	*Rpn10*	Proteasome 26S Subunit Ubiquitin Receptor, Non-ATPase 4	*chr1:151,254,709-151,267,479*
PSMD5	*KIAA0072, S5B*	Proteasome 26S Subunit, Non-ATPase 5	*chr9:120,815,496-120,842,951*
PSMD6	*Rpn7*	Proteasome 26S Subunit, Non-ATPase 6	*chr3:64,010,550-64,024,010*

PSMD7	*Rpn8*	Proteasome 26S Subunit, Non-ATPase 7	*chr16:74,296,814-74,306,288*
PSMD8	*Rpn12*	Proteasome 26S Subunit, Non-ATPase 8	*chr19:38,374,550-38,383,824*
PSMD9	*Rpn4*	Proteasome 26S Subunit, Non-ATPase 9	*chr12:121,888,732-121,918,297*
PSMD10	*Gankyrin, P28*	Proteasome 26S Subunit, Non-ATPase 10	*chrX:108,084,207-108,091,549*
PSMD11	*Rpn6*	Proteasome 26S Subunit, Non-ATPase 11	*chr17:32,444,379-32,483,319*
PSMD12	*Rpn5*	Proteasome 26S Subunit, Non-ATPase 12	*chr17:67,337,916-67,366,605*
PSMD13	*Rpn9*	Proteasome 26S Subunit, Non-ATPase 13	*chr11:236,966-252,984*
PSMD14	*Rpn11*	Proteasome 26S Subunit, Non-ATPase 14	*chr2:161,308,425-161,411,717*
PSMD15	*SEM1, DSS1, SHFM1*	SEM1 26S proteasome subunit	*chr7:96,688,762-96,709,846*
PSMD16	*Rpn13, ADRM1, GP110*	ADRM1 26S proteasome ubiquitin receptor	*chr20:62,302,093-62,308,862*

2 Supplementary Figures

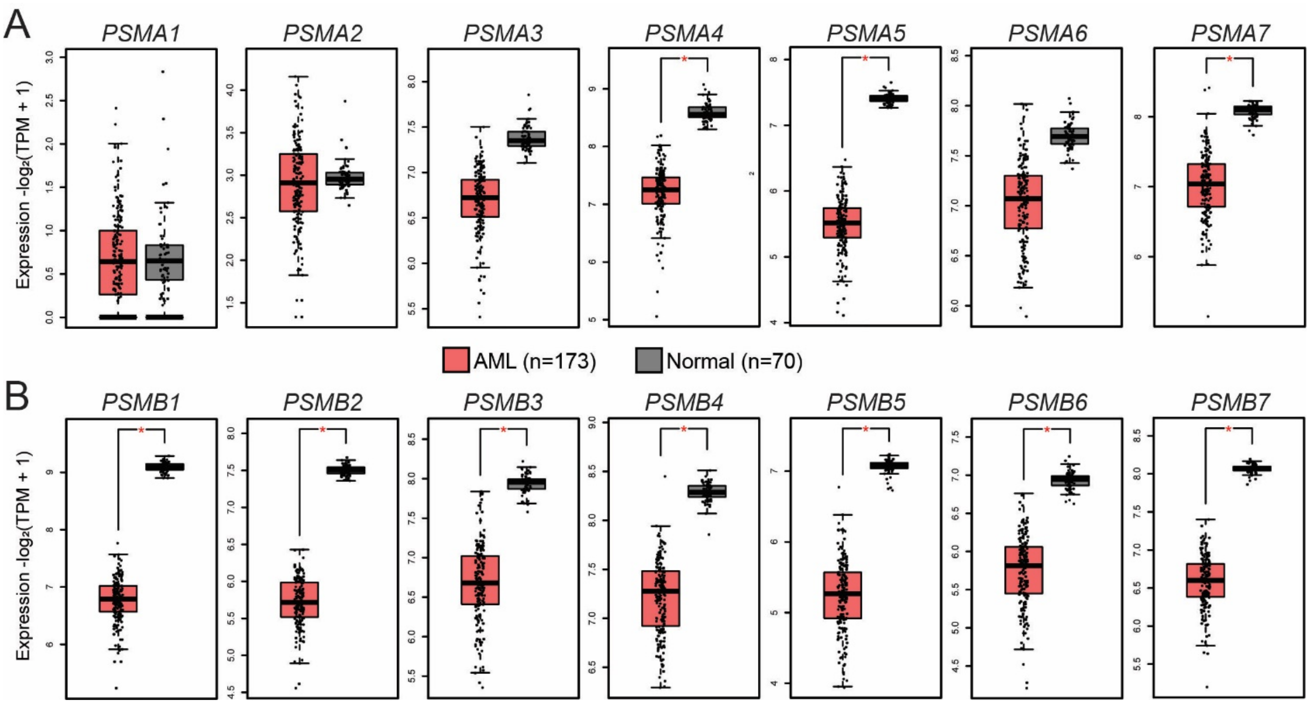

Supplementary Figure S1. Expression of mRNA encoding 20S proteasome subunits was downregulated in acute myeloid leukemia (AML) versus normal progenitor cells using GEPIA2. A-B. The box plots represent the expression of *PSMA1-7* (**A**) and *PSMB1-7* (**B**) mRNA comparing AML versus normal mononuclear cells (MNCs) from GEPIA2 (http://gepia2.cancer-pku.cn, accessed on May 26, 2023). Error bars represent standard error of the mean (SEM, *$p > 0.001$).

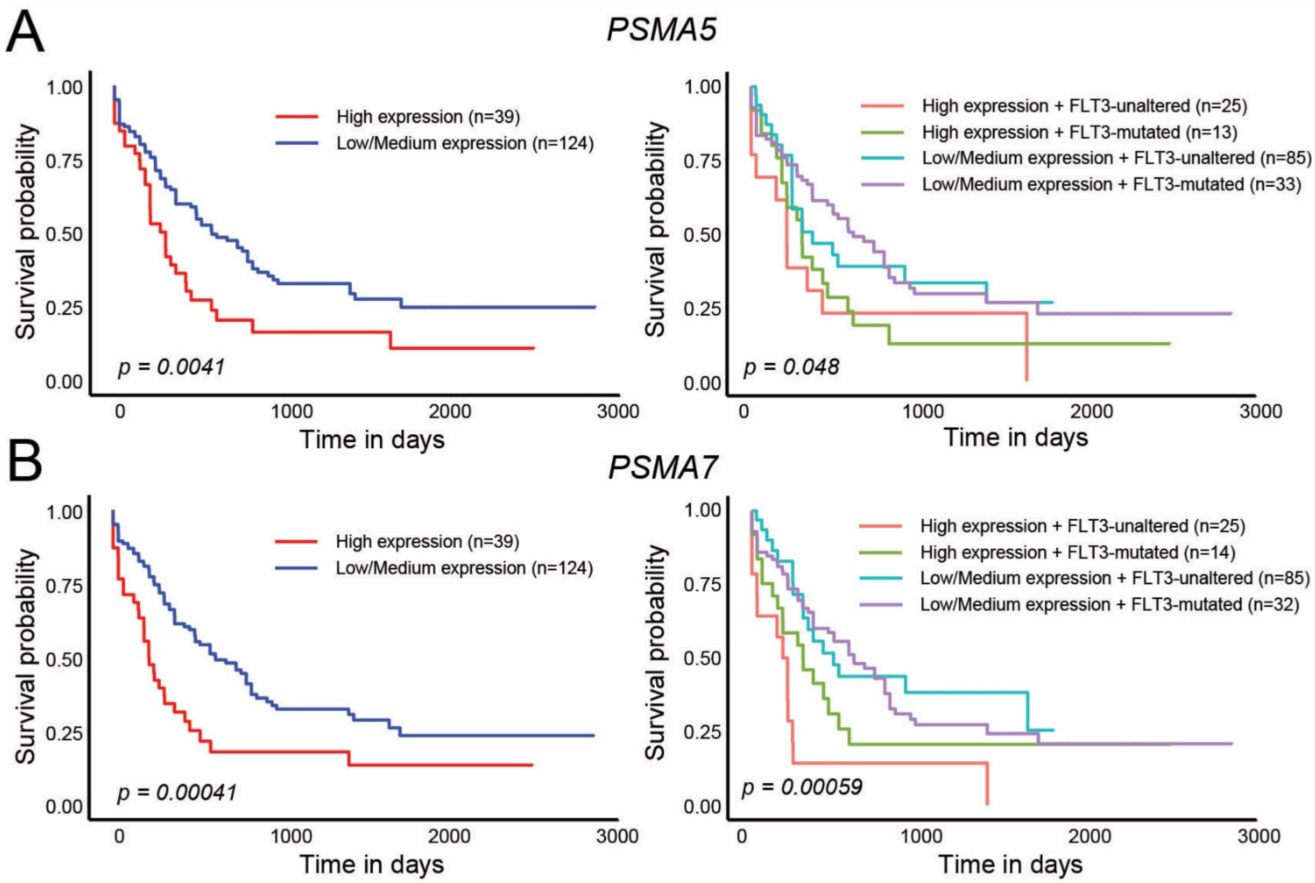

Supplementary Figure S2. High expression of mRNA encoding PSMA5 and PSMA7 of the 20S proteasome correlated with worse overall survival in AML. A-B. Kaplan-Meier curves show the effect of *PSMA5* (**A**) and *PSMA7* (**B**) mRNA expression on overall survival in AML when all subsets are considered (*left*) and in patients with mutations in the FMS-like tyrosine kinase 3 (*FLT3*) gene (*right*) from UALCAN (https://ualcan.path.uab.edu/, accessed on May 26, 2023).

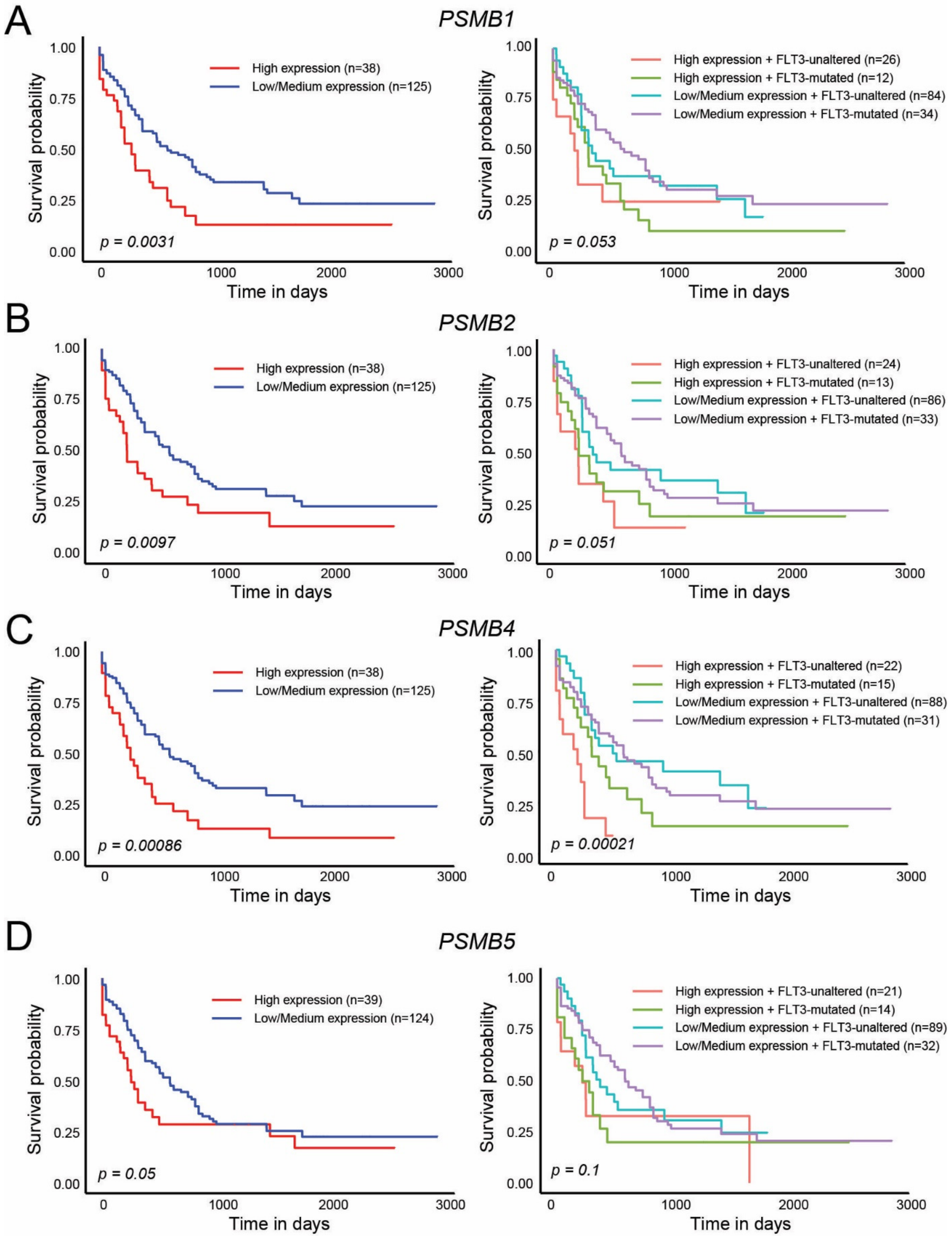

Supplementary Figure S3. High expression of mRNA encoding PSMB1, PSMB2, PSMB4, and PSMA5 of the 20S proteasome correlates with worse overall survival in AML. A-D. Kaplan-Meier curves show the effect of *PSMB1* (**A**), *PSMB2* (**B**), *PSMB4* (**C**), and *PSMB5* (**D**) mRNA expression on overall survival in AML comparing all subsets (*left*) and patients with FLT3 mutations (*right*).

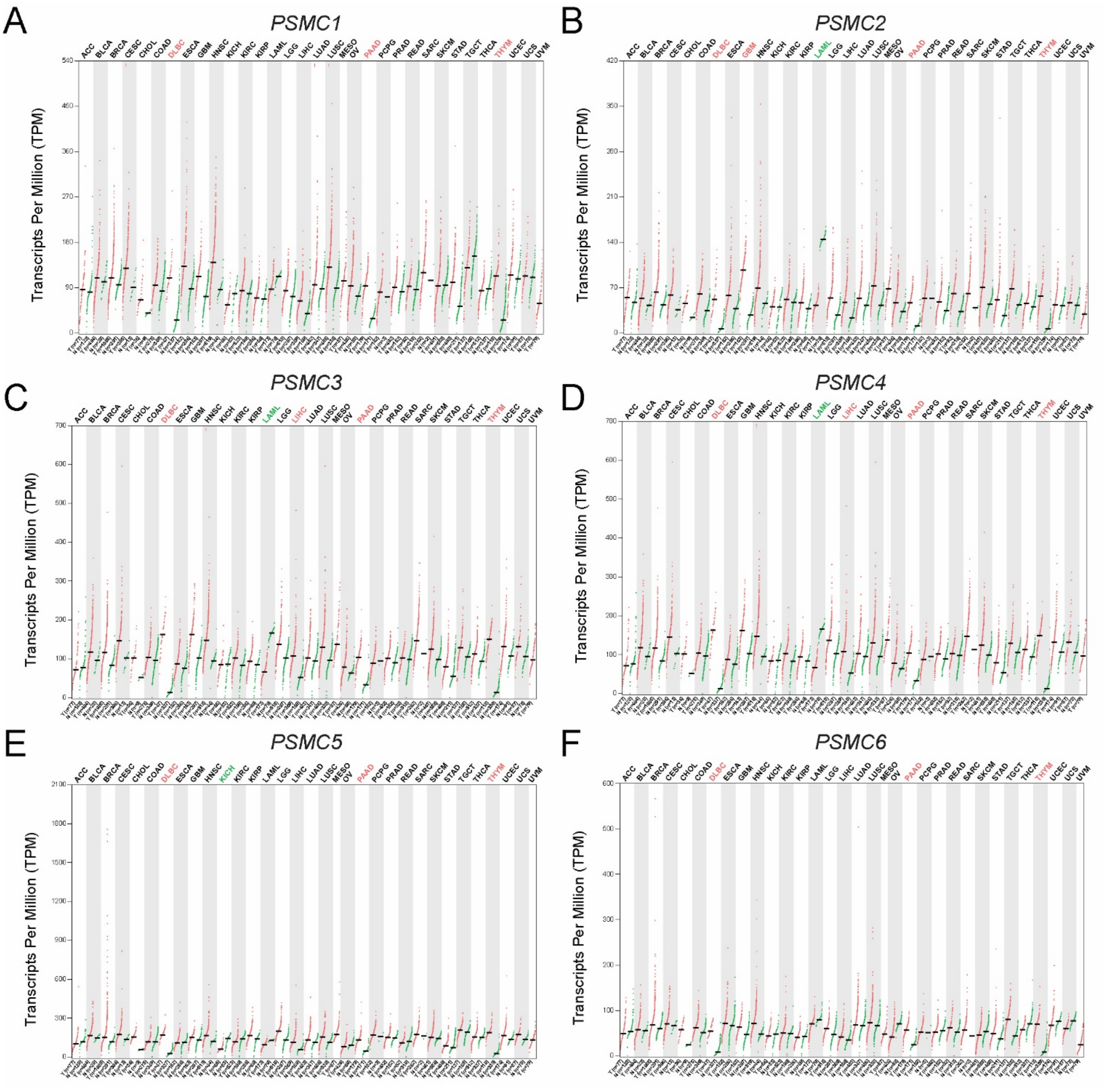

Supplementary Figure S4. Expression of mRNA encoding PSMC2, PSMC3, and PSMC4 were downregulated in AML versus normal progenitor cells using GEPIA2. A-F. Graphs show the expression of *PSMC1* (**A**), *PSMC2* (**B**), *PSMC3* (**C**), *PSMC4* (**D**), *PSMC5* (**E**), and *PSMC6* (**F**) mRNA comparing tumor versus normal tissue for all of the cancers available in the database (http://gepia2.cancer-pku.cn, accessed on January 12, 2023). The red font names symbolize the cancers that are significantly upregulated, whereas the green font names are of those cancers that are significantly downregulated.

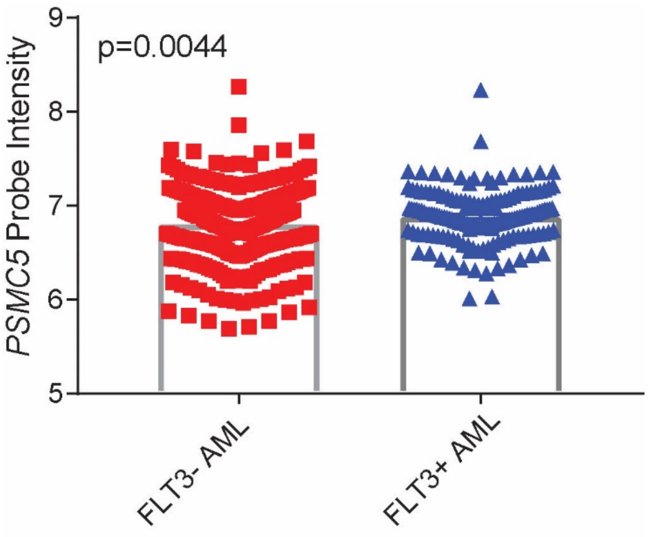

Supplementary Figure S5. *PSMC5* mRNA expression is upregulated in AML patients with mutated versus wild-type FLT3. The bar graph shows *PSMC5* mRNA expression comparing AML patients with mutated versus wild-type FLT3. *PSMC5* was significantly upregulated in FLT3-mutated AML (p=0.0044).

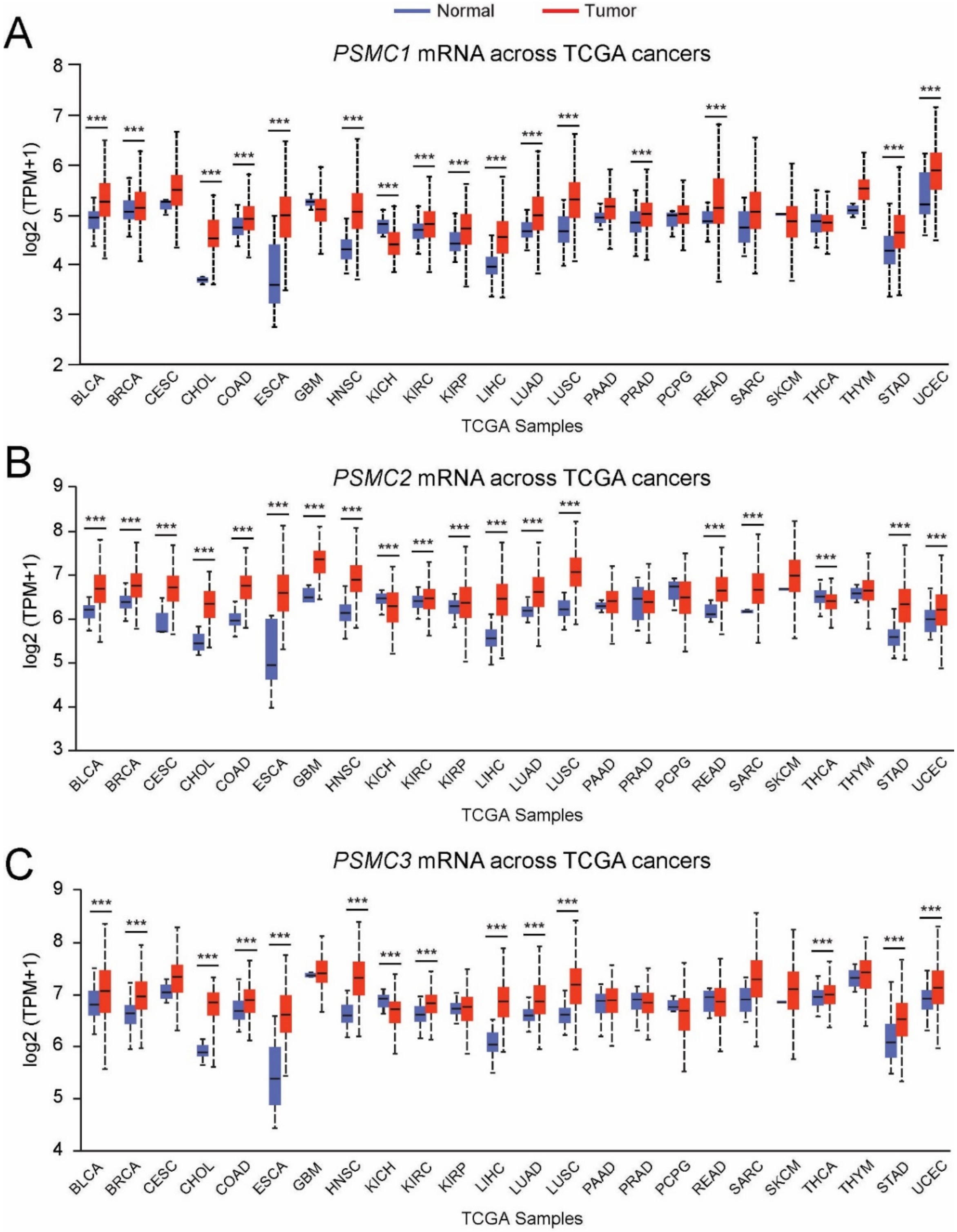

Supplementary Figure S6. *PSMC1-3* mRNA expression is upregulated in several solid tumors compared with normal tissue. A-C. The box plots represent *PSMC1* (**A**), *PSMC2* (**B**), and *PSMC3* (**C**) mRNA expression in tumor versus normal samples from The Cancer Genome Atlas (TCGA). ***$p < 0.001$.

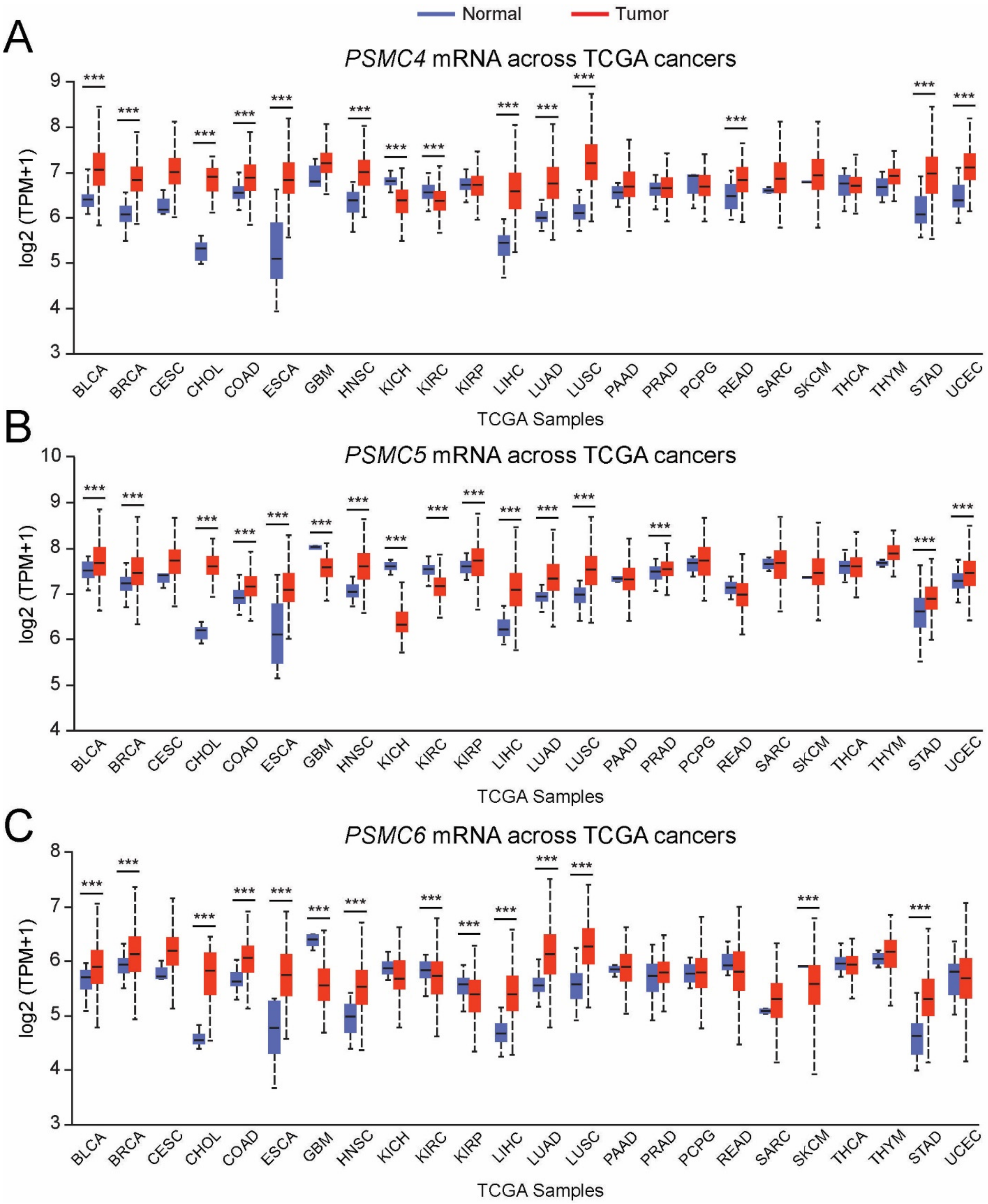

Supplementary Figure S7. *PSMC4-6* mRNA expression is upregulated in several solid tumors compared with normal tissue. A-C. The box plots represent *PSMC4* (**A**), *PSMC5* (**B**), and *PSMC6* (**C**) mRNA expression in tumor versus normal samples from TCGA. ***$p < 0.001$.

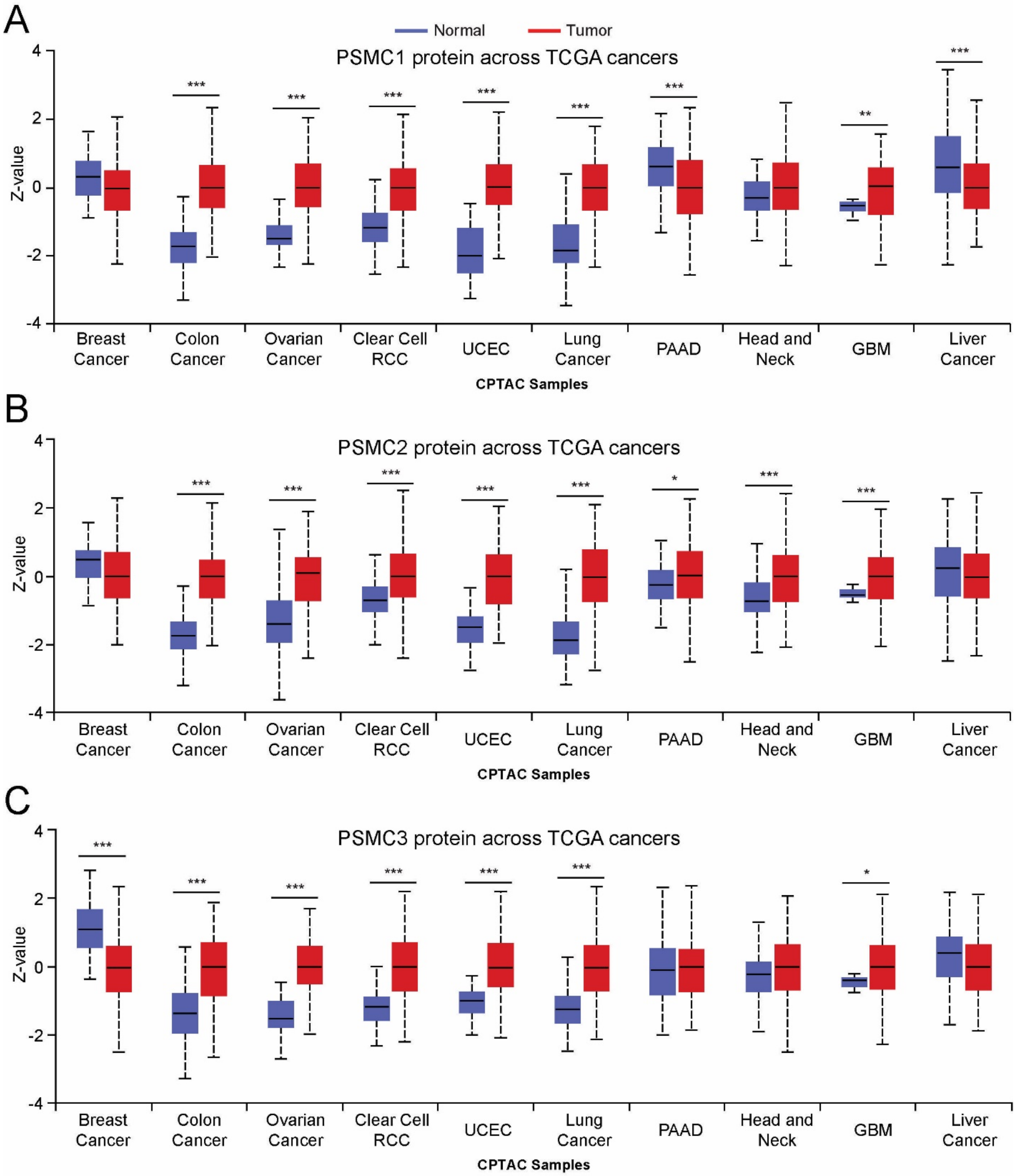

Supplementary Figure S8. PSMC1-3 protein expression is upregulated in several solid tumors compared with normal tissue. A-C. The box plots represent PSMC1 (**A**), PSMC2 (**B**), and PSMC3 (**C**) protein expression in tumor versus normal samples from The National Cancer Institute's Clinical Proteomic Tumor Analysis Consortium (CPTAC). *$p < 0.05$; **$p < 0.01$; ***$p < 0.001$.

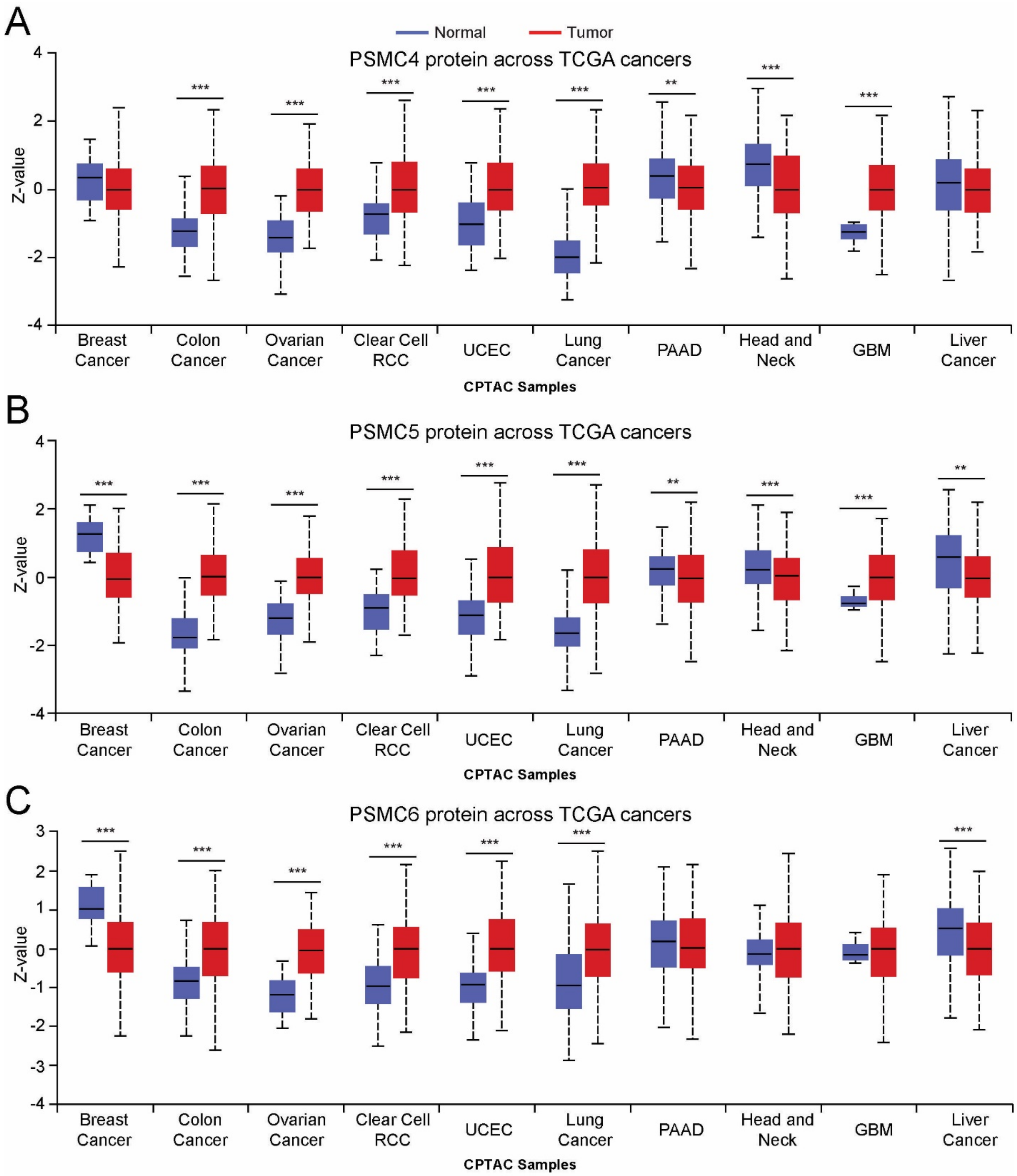

Supplementary Figure S9. PSMC4-6 protein expression is upregulated in several solid tumors compared with normal tissue. A-C. The box plots represent PSMC4 (**A**), PSMC5 (**B**), and PSMC6 (**C**) protein expression in tumor versus normal samples from The National Cancer Institute's CPTAC. **p <0.01; ***p < 0.001.

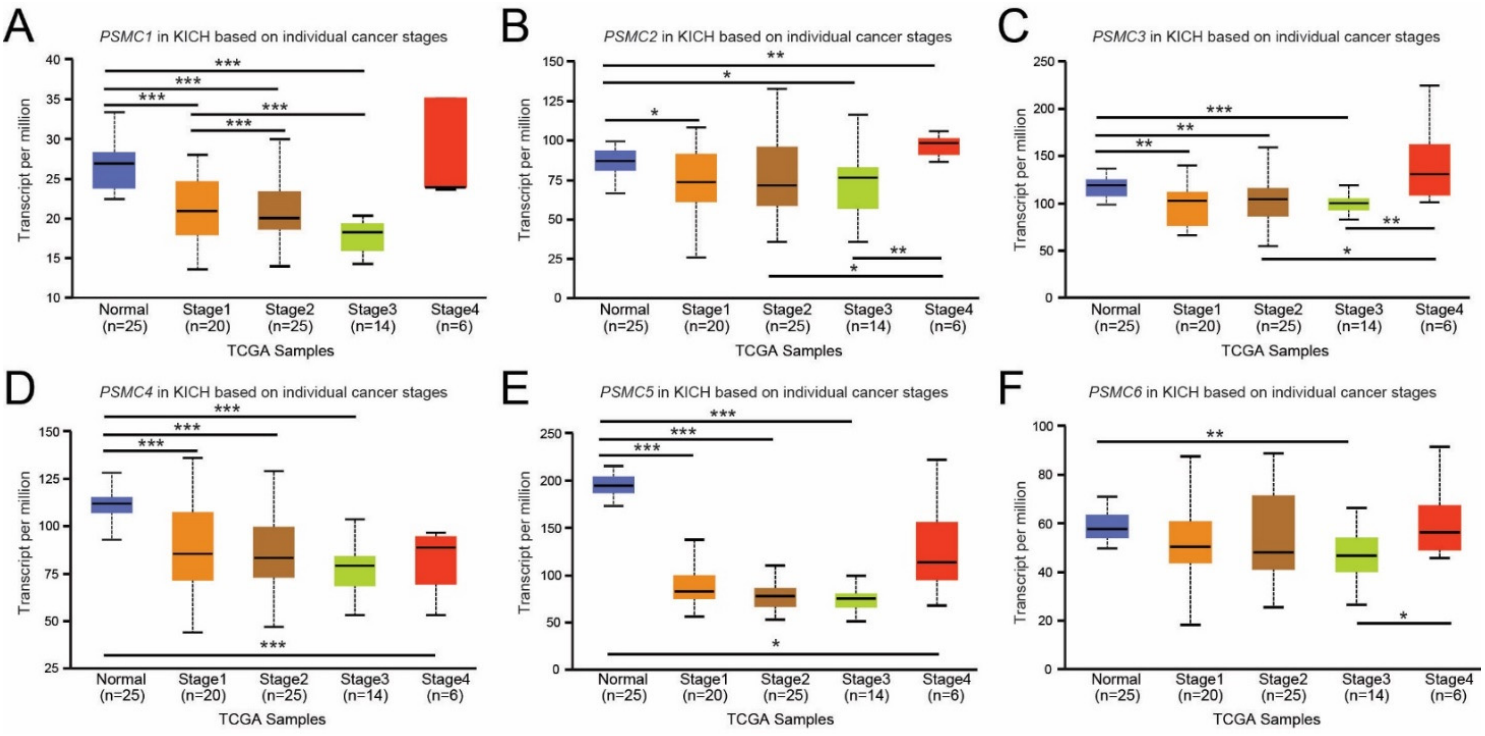

Supplementary Figure S10. *PSMC1-6* expression in kidney chromophobe (KICH) by stage of disease. A-F. The box plots represent *PSMC1* (**A**), *PSMC2* (**B**), *PSMC3* (**C**), *PSMC4* (**D**), *PSMC5* (**E**) and *PSMC6* (**F**) mRNA expression in stages 1-4 of KICH compared with normal samples from TCGA. *$p < 0.05$; **$p < 0.01$; ***$p < 0.001$.

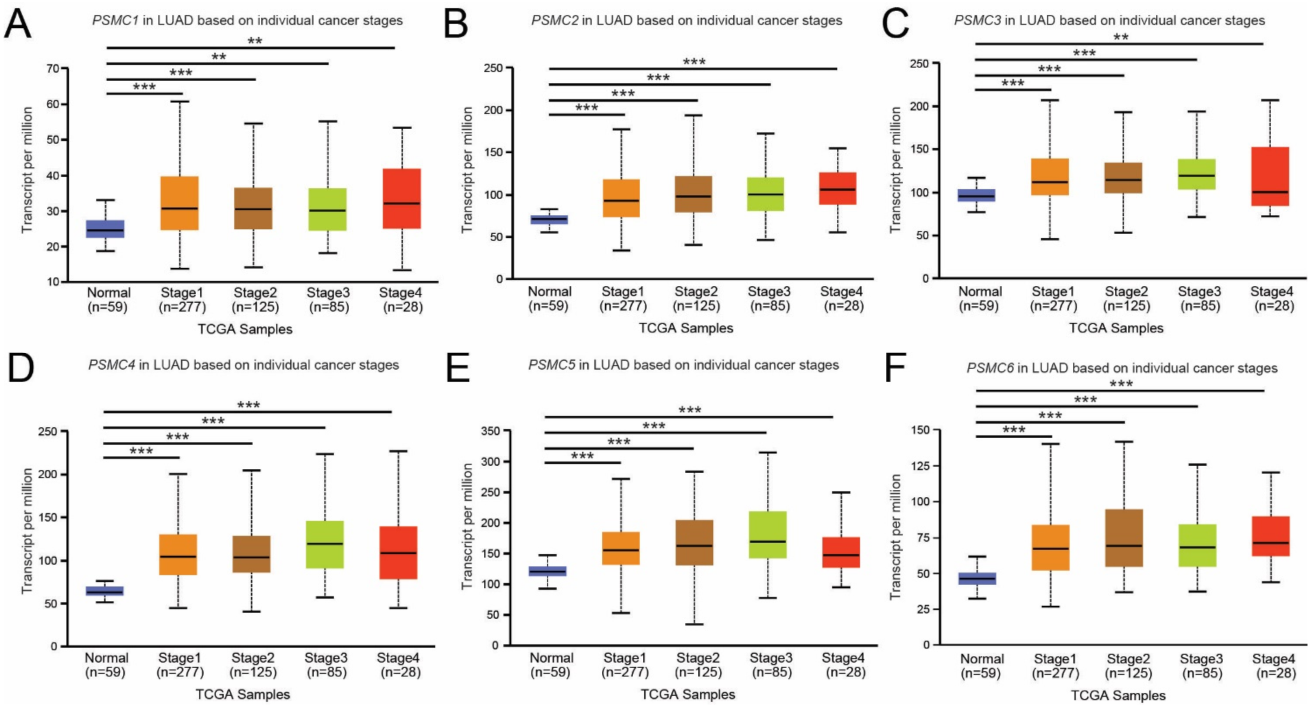

Supplementary Figure S11. ***PSMC1-6* expression in lung adenocarcinoma (LUAD) by stage of disease. A-F.** The box plots represent *PSMC1* (**A**), *PSMC2* (**B**), *PSMC3* (**C**), *PSMC4* (**D**), *PSMC5* (**E**) and *PSMC6* (**F**) mRNA expression in stages 1-4 of LUAD compared with normal samples from TCGA. **p <0.01; ***p < 0.001.
